# Modulation of interbrain synchrony by emotional valence and maternal presence in mother–child dyads: neural links to empathy and attachment

**DOI:** 10.1038/s41598-026-43086-7

**Published:** 2026-03-16

**Authors:** Inês Rodrigues, João Pereira, Diana Costa, Rita Correia, Marco Simões, Bruno Direito, Pascal Vrtička, Teresa Sousa, Miguel Castelo-Branco

**Affiliations:** 1 CIBIT – Coimbra Institute for Biomedical Imaging and Translational Research, University of Coimbra, Coimbra, Portugal; 2 ICNAS – Institute for Nuclear Sciences Applied to Health, University of Coimbra, Coimbra, Portugal; 3 Faculty of Medicine, FMUC – Institute of Physiology, University of Coimbra, Coimbra, Portugal; 4 CISUC - Centre for Informatics and Systems of the University of Coimbra, Coimbra, Portugal; 5 LASI - Intelligent Systems Associate Laboratory, Guimarães, Portugal; 6 Department of Informatics Engineering, University of Coimbra, Coimbra, Portugal; 7 Centre for Brain Science, Department of Psychology, University of Essex, Colchester, UK; 8 Maastricht Brain Imaging Center, Department of Cognitive Neuroscience, Maastricht University, Maastricht, The Netherlands

**Keywords:** fNIRS, Hyperscanning, Pre-adolescence, Mother-child, Social neuroscience, Affective neuroscience, Neuroscience, Psychology, Psychology

## Abstract

Interbrain synchrony (IBS) is thought to reflect the flow of information between individuals and interpersonal mechanisms like empathy and bonding. Previous research suggests that IBS varies according to different factors, including interpersonal bond type, individual characteristics, emotional valence and *physical* presence/absence. This study investigated how IBS in mother-child dyads varies during *imagined* emotional situations, focusing on valence and imagined presence/absence of the mother. We used functional near-infrared spectroscopy (fNIRS) hyperscanning to measure IBS over the right prefrontal cortex, dorsolateral prefrontal cortex and temporoparietal junction in 38 mother-child dyads (child age: 10–14 years). Results showed that IBS differed across valence only when participants imagined the mother being present, with higher IBS in negative situations than in positive ones. Furthermore, in scenarios imagined with the mother present, IBS was associated with the mother’s personal distress —negative correlation in positive scenarios, and positive correlation in negative scenarios—and with children’s secure base support scores—negative association in the frontopolar cortex. These findings suggest that mothers and children perceive being together differently, depending on emotional valence, social context and individual differences. Overall, our results suggest that IBS reflects dyadic emotional processing and highlight associations with individual traits in dyadic neural synchrony.

**Supplementary Information:**

The online version contains supplementary material available at 10.1038/s41598-026-43086-7.

## Introduction

Bio-behavioural synchrony (BBS) refers to the temporal alignment of behaviours, physiological and neurobiological processes – including heart rate responses, hormonal modulations and brain activity – between people during and shortly after social interaction^1^. Amongst those, interbrain synchrony (IBS) is thought to reflect the flow of information between individuals in general^2^—but particularly interpersonal mechanisms such as cooperation, empathy, attachment and co-regulation^3^—and is usually stronger the closer/more intimate the interpersonal bonds are^1,4,5^. That said, IBS can also vary within a given interpersonal bond type as a function of both interaction and relationship quality, depending on, e.g., individual differences in attachment^3^. Furthermore, while increased IBS is often interpreted as beneficial for social interaction quality and outcomes, it is important to acknowledge that the adaptiveness of increased IBS depends on the specific social interaction context and individual differences in personality traits, as reflected by the Theory of Flexible Modality^6^.

Hyperscanning is a neuroimaging approach that involves the simultaneous acquisition of brain activity from two or more participants to derive a measure of IBS^7^. Hyperscanning studies vary with interactivity level, ranging from co-presence to two-way or group interactions. In co-presence studies, participants’ brain activity is simultaneously measured while they share experiences that do not require direct interactions (e.g., passive film watching). Conversely, two-way or group interactions require an active exchange between participants (e.g., interactive games like puzzles^3^. While different methodologies can be used for hyperscanning, such as electroencephalography (EEG) or functional magnetic resonance imaging (fMRI)^8^, fNIRS is the most frequently employed^9^. Being more affordable than fMRI, fNIRS also allows for more naturalistic, experimental setups^9,10^. Furthermore, fNIRS has better spatial resolution and is more robust to motion artefacts than EEG^10^. The present study aimed to study IBS in mother-child dyads using a co-presence emotional imagery task.

Co-presence hyperscanning studies enable the investigation of IBS as a reflection of how similarly participants experience external stimuli and how similar their neural processing of these experiences is. For example, fNIRS hyperscanning was used to study mother-child dyads while they watched animation videos of varying valence together. While no association was found between IBS and video valence, higher maternal-reported stress was linked to lower IBS, highlighting the importance of mothers’ mental well-being in mother-child shared experiences^11^. In another study, adult dyads watched positive and neutral videos on individual monitor screens, sitting across from each other. Here, IBS was higher for positive than neutral videos, suggesting that affective external stimuli are processed more similarly than neutral information^12^.

While increased IBS may occur when participants are exposed to the same external stimuli, their experience of these stimuli can differ depending on social context. For example, the effect of co-presence on IBS was assessed while co-parent dyads listened to stimuli of different emotional valences in the same or separate rooms. IBS for positive and neutral stimuli (but not for negative) was higher when both parents listened to the stimuli in the same room (*versus* in separate rooms). These findings suggest that co-presence and valence influence IBS. When together, dyads tended to perceive auditory stimuli more similarly, potentially facilitating behavioural coordination. The absence of increased IBS during shared exposure to negative stimuli (i.e., infants’ cry) may reflect adaptive caregiving, with co-parents responding differently to stress^13^. Another study investigated the effect of co-presence during a mathematical task, with adult-adult dyads solving tasks on individual computers, either side-by-side separated by a visual barrier or sitting in separate rooms. Here, the adjacent presence of another person yielded increased IBS, possibly reflecting participants’ awareness of each other despite the lack of visual contact^14^. Together, these findings suggest that co-presence influences similarity in the perception of external stimuli and problem solving, often associated with an increase in IBS compared to physical separation. However, this effect may vary depending on factors such as stimulus valence and personal traits.

It has been observed that individual participant characteristics might be related to IBS variations^3^. Besides the association between mother-child IBS and mothers’ stress previously mentioned^11^, IBS during a parent-child video co-watching task has also been associated with mothers’ attachment traits, where preliminary results show lower IBS when mothers had higher attachment anxiety^15^. Another study investigated the link between IBS and attachment as parent-child dyads completed tangram puzzles cooperatively *versus* individually. In the cooperation condition, mother-child dyads in which mothers with insecure (*versus* secure) attachment showed higher IBS, suggesting that mother-child dyads with insecure mothers need to make an extra effort to be in tune with one another^16^. In adult-adult dyads, it was observed that IBS was positively correlated with dyadic self-reported empathy traits during competition and cooperation tasks, suggesting that dyads with higher empathy scores exhibited more similar brain activation patterns in both contexts^17^. Overall, these studies indicate that, regardless of the level of interactivity between the participants/dyads, factors such as individual and dyadic traits, as well as the quality of their relationship, may be reflected in IBS.

In this fNIRS hyperscanning study, mother-child dyads engaged in a co-presence mental imagery task, where the research hypothesis was based on the imagined presence *versus* absence in situations of distinct valence. Unlike previous studies that probed associations between IBS and the *physical* presence *versus* absence of participants, our work introduces a novel design to investigate IBS in imagined scenarios. To do so, we designed a mental imagery task during which parents and children sat in the same room—without visual contact—and imagined experiencing positive, negative, and neutral situations targeting the child, either while imagining being together or apart. Mental imagery was chosen based on previous research showing that mental imagery is effective in evoking emotions^18–20^ and that imagined support influences emotional experiences^21^. Furthermore, we opted to assess mother-child dyads with children in the adolescent age range (ages 10–14), as adolescents, like younger children, still rely on their parents for support. Particularly, they usually report more positive affect when their parents are present, regardless of the situation’s emotional valence^22^. The variation of adolescents’ affective experiences based on their caregivers’ presence provides a compelling framework to investigate potential differences in IBS as a function of caregiver availability.

We measured dyadic brain activity in the right temporoparietal junction (rTPJ), dorsolateral prefrontal cortex (rdlPFC) and right frontopolar cortex, brain areas commonly investigated in fNIRS hyperscanning studies^11,23,24^. The TPJ is associated with mentalising and theory of mind^25–27^, while the dlPFC and frontopolar areas are implicated in emotion processing and regulation as well as attention^25–27^.

Given the association between individual differences in certain personality traits and IBS, we also aimed to study the relationship between IBS and mothers’ empathy and children’s attachment (to the mother). These traits were selected given empathy’s role in understanding others’ emotions^28,29^ and attachment as a reflection of the child-caregiver relationship quality (reviewed in ref.^30)^, which is also linked to IBS^3,16^.

We hypothesised that mother-child IBS during imagined experiences differs as a function of both social condition (i.e., imagining being together *versus* apart) and emotional valence. Additionally, we hypothesised that IBS would be correlated with mothers’ empathy and children’s attachment, reflecting both the mothers’ capacity to understand their children’s emotions and the mother-child relationship quality^3^, respectively.

## Methods

### Participants

We recruited 41 mother-child dyads (81 participants; one mother came twice with both her sons), with children’s ages ranging from 10 to 14 years. All children participated with their biological mother, and participants were healthy with no current or previous substance abuse. Inclusion criteria furthermore included normal development, no history of brain trauma or psychiatric disorders, and no current diagnosis of psychological disorders. One dyad was excluded because the mother was not feeling well and did not complete the task, two other dyads were excluded due to bad fNIRS data quality (see below for more details), and due to technical issues, one pair of participants only had data from half the task (one run per social condition, see below for more details). The final sample, therefore, included data from 38 dyads (19 girls, M_children’s age_ = 12.55 years, SD_children’s age_ = 1.03; M_mothers’age_ = 46.53 years, SD_mothers´age_ = 4.30). All participants were native Portuguese speakers, 93% were right-handed (self-reported), 21% of the children were an only child, and 92% of the mothers had a University degree while the remaining 8% completed high school. The study was approved by the Ethics Committee of the Faculty of Medicine at the University of Coimbra (reference CE-099/2020), and all participants provided written informed consent (i.e., mothers also signed the consent forms on behalf of their children). All methods were performed in accordance with the relevant guidelines and regulations.

### Procedure and set-up

Mother-child dyads were invited to the laboratory for a two-hour visit. The task was explained in detail, and participants were trained using a PowerPoint presentation with a real task example of each valence. Participants filled out individual difference scales (lasting approximately 20 min) at the beginning or end of their visit. After the fNIRS cap fitting and electrodermal activity (EDA) electrodes placement (see Supplementary material S1 and S3 for EDA analyses and results), participants completed an emotional imagery task. Participants completed the task while seated back-to-back, with 170 cm separating their desks, ensuring they could not see each other during the task. They faced individual computer screens (distance to screen = 55 cm) where they could see the stimuli and used a keyboard to rate the stimuli (see Fig. 3a)

### Task

**Fig. 1:**
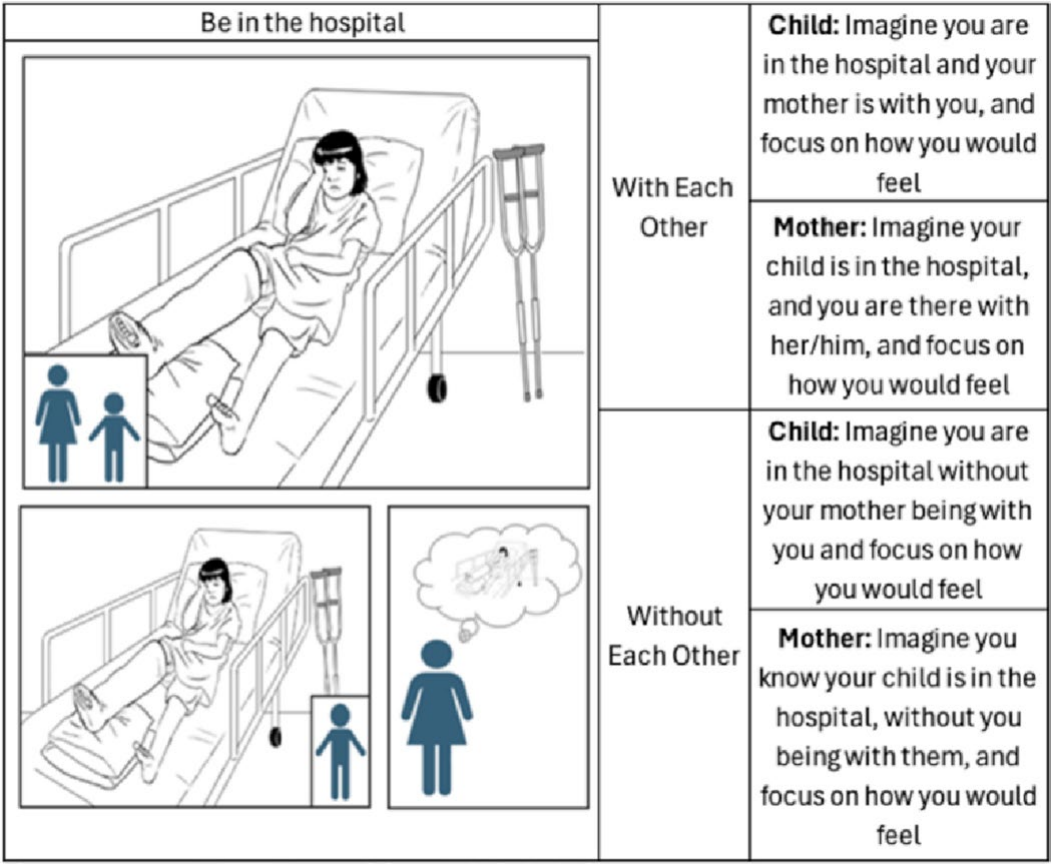
Schematic representation of the emotional imagery task, with the example of a negative situation (picture 14 from the PiSCES database) labelled as “Be in the hospital”. The top row depicts the “with each other” condition, and the bottom row represents the “without each other” condition. The right panel contains the corresponding instructions.

Participants completed an emotional imagery task during which they were both asked to imagine scenarios where the child always was the primary target of emotional situations. The presented scenarios had three different valences (i.e., positive, negative, or neutral), and the participants were asked to imagine how the situation would make them feel in two different social conditions: *with each other versus without each other*, i.e., together *versus* apart. In the *with each other* condition, participants were told to imagine the mother acting as an emotional support figure without being an active target of the action. Therefore, both mother and child should imagine being together while the child is going through the situation, and each participant should focus on how that situation makes them feel. In the *without each other* social condition, participants were told to imagine that the child was living the situation alone, without the mother being present. More specifically, the mother should imagine the child living the situation without her being there (and focus on how that made her feel), while the child should focus on how they feel living the presented situation without the mother being with them. For example, one scenario involved the child playing tennis with the label “Win a game” (picture ID 142 in the supplementary materials Figure S1). Accordingly, participants were instructed to imagine the child playing a game they enjoyed and to focus on how their win would make them feel. Specifically, in the *without each other* condition, the mother was asked to imagine she knew her child won a game while she was not present and to focus on how the situation made her feel. The child was asked to imagine winning a game without their mother present and to focus on how it made them feel. For more information and further examples, see Fig. 1 and Supplementary Figure S1.

**Fig. 2:**
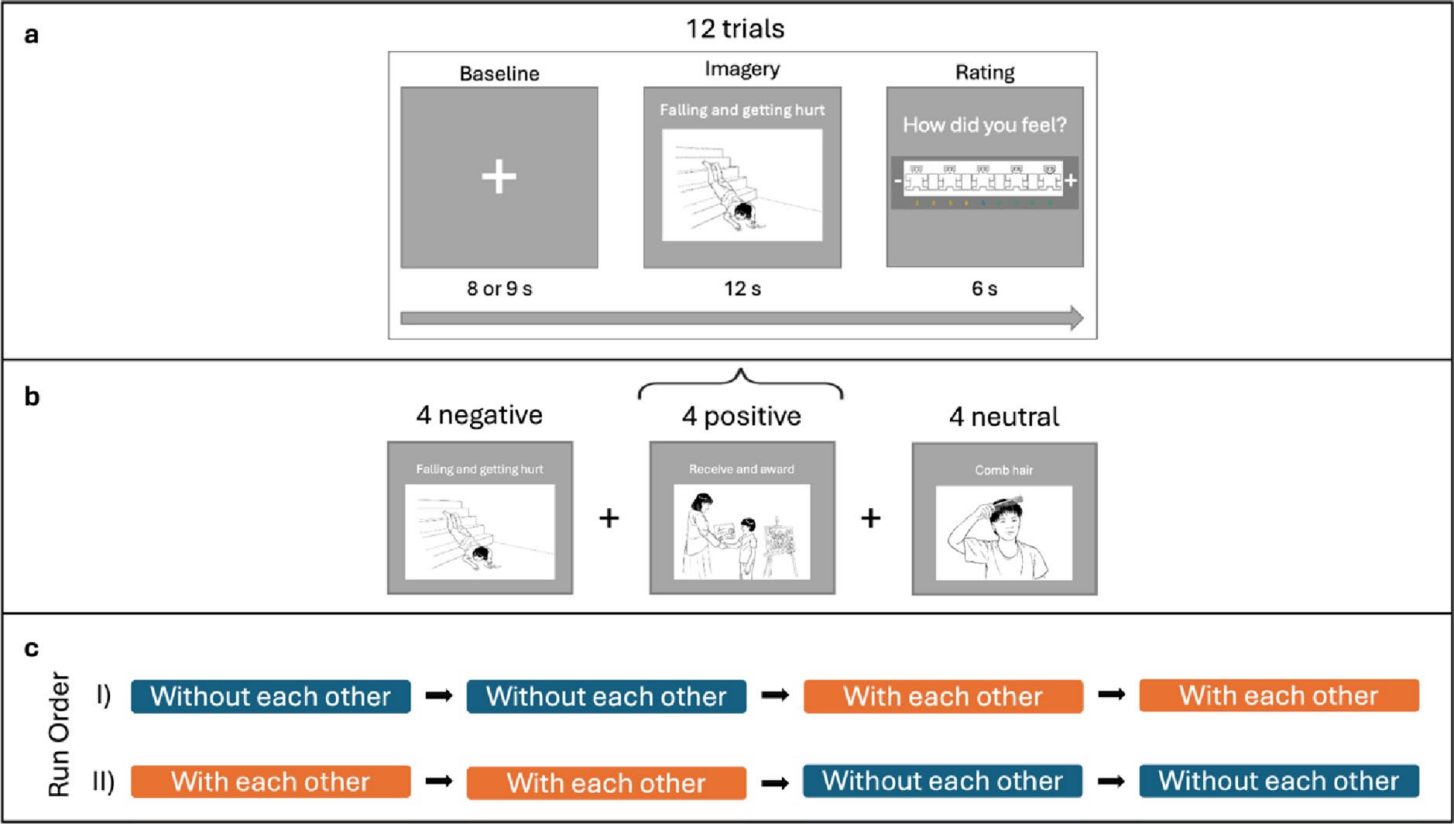
(**a**) Each trial began with a fixation cross (jittered between 8 to 9s), followed by a 12s imagery period and ending with a subjective rating using a SAM scale within a 6s time window. In total, there were 12 trials per run, with the first baseline in each run lasting for 20s. (**b**) Each run consisted of four situations of each valence - negative, positive, and neutral – presented in a semi-random order. (**c**) Task sequence. Each dyad completed four runs, two runs in the with each other and two runs in the without each other social condition. The sequence of the runs followed either the order presented in I or in II and was counterbalanced across the participants.

The full experimental sequence included a series of trials comprising an imagery period that was preceded by a white fixation cross (visual angle = 4.96° × 2.79°) and was followed by a subjective valence rating (Fig. 2). There were four runs (2 focused on the imagined *with each other* condition and other on the 2 imagined *without each other* condition), each run consisting of twelve trials (4 positive, 4 negative, and 4 neutral imagery periods) presented in a semi-random order, all imagined within the same social condition, resulting in 8 imagery periods for each valence within each social condition. Each imagery period was preceded by a jittered baseline during which participants were instructed to look at a fixation cross (Fig. 2B). Mother and child always viewed the same stimuli at the same time, and the order of the social conditions was counterbalanced between dyads. Images (visual angle = 33.77° × 13.89°) were sourced from PiSCES^31^, an open-source database containing black-and-white drawings of social and non-social situations with positive, negative, and neutral valence. Each image was paired with a white label (visual angle = 4.19°) that instructed participants regarding the imagined scenario (all images and corresponding labels can be found in the supplementary materials Figure S1). Stimuli were presented using *PsychoPy* (version 2022.2.4), and synchronisation between task events and data acquisition was achieved via event markers transmitted through the parallel port.

### Behavioural measures

As behavioural measures, we used participants’ individual subjective valence ratings for the imagined situations, provided using the pictorial 9-point SAM Manikin scale^32^. In the SAM Manikin scale, 1 represents the most negative feeling, 9 the most positive feeling and 5 a neutral state (Fig. 2A). For each participant, we calculated the average score for the situations of each valence in each social condition (i.e., 3 valence x 2 social condition factorial design), resulting in 6 behavioural scores.

Additionally, we calculated two behavioural dyadic measures: dyadic average subjective valence rating and dyadic differences in subjective valence ratings. Dyadic average subjective ratings represented the average value between the children’s average valence rating and the mothers’ average valence ratings for each valence in each social condition. Differences in average valence ratings were calculated as the difference in valence ratings between mothers and children as the absolute value of the subtraction of the average children’s rating from the mothers’ average rating for each valence in each social condition. For each dyad, we thus derived 6 additional values representing the averages and similarity in mothers’ and children’s subjective valence ratings.

### Psychological measures

#### Mothers

The Interpersonal Reactivity Index (IRI)^33,34^ is a self-report empathy scale for adults. It has four subscales: perspective taking (PT; adopting the other person’s perspective), empathic concern (EC; tendency to feel concern for others), personal distress (PD; feeling of self-unease or self-discomfort when faced with another’s distress), and fantasy—not used for this study, as it is mainly related to one’s capacity to tap into the emotions and actions of fictional characters. Higher scores represent a higher use of that empathy dimension.

#### Children

We used the Security Scale Questionnaire (SSQ)^35,36^ as a self-report scale to evaluate children’s perception of their attachment to their parents. The SSQ has two subscales: Safe Haven Support (SHS; children’s use of the parent as a safe haven when in distress) and Secure Base Support (SBS; children’s perception of parents as a secure base during exploration of challenges of both a physical and social nature). Higher scores represent greater attachment security. For this study, only the items related to the mother were considered.

### fNIRS

#### Optode placement and data collection

**Fig. 3:**
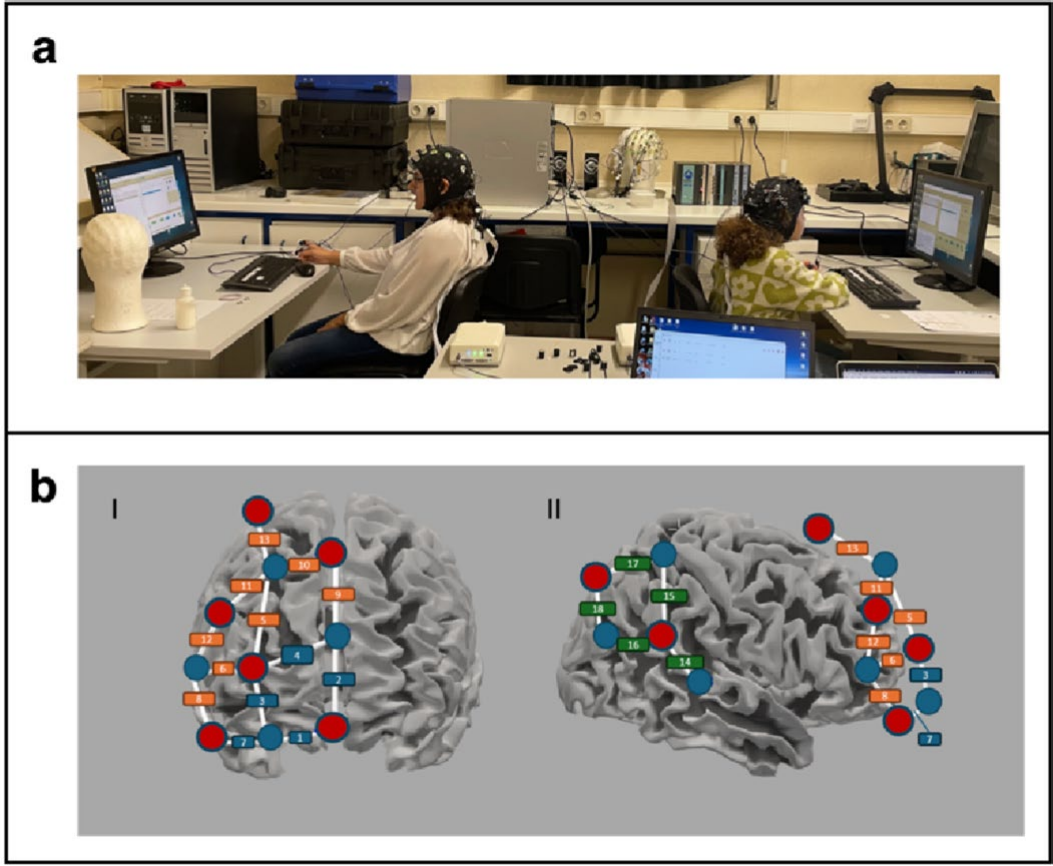
(**a**) Experimental set-up with participants facing individual computer screens. The task was performed with participants sitting back-to-back, preventing them from seeing each other during the task. (**b**) Illustration of fNRIS optode and channel placement. The anterior view is shown on the left (I), and the right lateral view is shown on the right (II). Red dots represent emitters, blue dots represent detectors, and blue circles around the emitters (red dots) represent short-distance channels. Channels formed by emitter-detector pairs are indicated by numbers, where blue squares represent the frontopolar area (I and II), orange the dlPFC (I and II), and green the TPJ (II) ROIs.

Two fNIRS devices (NIRSport2 continuous wave fNIRS system, NIRx Medical Technologies, Berlin) were used to simultaneously measure brain activity from both participants with the Aurora fNIRS acquisition software in hyperscanning mode (Brain Innovation, the Netherlands) at a sampling rate of 10.17 Hz. Optodes were placed over the right frontopolar cortex, rdlPFC and rTPJ with 8 standard-distance emitters, 7 standard-distance receptors and 8 short-distance detectors, resulting in 18 standard-distance channels placed over the three regions of interest - ROIs (5 frontopolar, 5 rTPJ and 8 rdlPFC) and 8 short-distance channels (one for each emitter). Optode placement and ROI grouping (based on channel specificity) were determined using the fOLD^37^ toolbox (version 2.2) (Fig. 3b and Table S1).

#### fNIRS data quality assessment and pre-processing

fNIRS data were pre-processed using Sartori 1.8 (Brain Innovation, the Netherlands). After raw data conversion to optical density, data quality was assessed by calculating the Scalp Coupling Index (SCI) for each run, using a 0.75 SCI value threshold as the exclusion boundary^38^. If a channel had at least one (out of two) runs with good data quality for each social condition (with/*without each other*), data from that channel were included. Accordingly, if there was no good quality data for at least one run in each social condition, all data from that channel was excluded for that participant and, consequently, the entire dyad. If a dyad had less than two good channels per ROI, the dyad was excluded from the analysis. Following this procedure, roughly 23% of channels were excluded from IBS analysis across dyads.

Pre-processing steps involved Temporal Derivative Distribution Repair (TDDR) motion correction (with restoration of high frequencies) and spike removal, short-channel regression by fitting a general linear model with the highest correlated short-channel for each channel signal as a regressor, using the highest correlated short-channel^39–41^, and temporal filtering (High-pass (Butterworth) = 0.01 Hz; Low pass = 0.50 Hz to remove slow drift noise and high-frequency noise, such as heart rate artifacts, from the fNIRS signal). The data were then converted from optical density data to concentration changes in oxygenated (HbO) and deoxygenated (HbR) haemoglobin and normalized (z-transformation).

#### Interbrain synchrony analysis

IBS was subsequently derived using wavelet transform coherence (WTC) analysis, implemented in MATLAB (version R2017b) and based on the Morlet wavelet^42^. WTC was calculated for each run between matching channels for each mother-child dyad. Coherence values were then averaged for each imagery period. More specifically, to align with the 12-second duration of the imagery period and to account for the hemodynamic response delay, coherence was averaged over 12-second windows beginning 4-second after imagery stimulus onset and ending 4-second after imagery stimulus offset^43,44^. This analysis was conducted across the 0.15–0.32 Hz frequency band (i.e., 3 to 6.5 period seconds). The frequency band of interest was chosen as a function of the imagery period length to ensure at least two to three full oscillation cycles at each signal frequency^45^. All coherence values outside the cone of influence were subsequently removed. In the end, each dyad had a total of 6 coherence values per included channel (3 valences x 2 social conditions), representing the average coherence value of all the imagery periods of each valence in each social condition.

## Statistical analyses

All statistical analyses were performed using RStudio (version 2023.12.1 + 402).

### Behavioural measures

We analysed subjective emotion valence ratings with a non-parametric ANOVA model, after testing data normality with the Shapiro-Wilk test, using the ARTool package^46^ which enables running ANOVAs on non-parametric data by applying the Aligned Rank Transform to the data beforehand. The model (model 1 specified in the supplementary material) included valence, social condition and subject (mother/child) as main and interacting factors. Participant ID was added as a random intercept. False Discovery Rate (FDR)^47^ was used to correct for multiple comparisons in *post hoc* analyses.

### Psychological measures

Means and standard deviations were calculated for all the subscales of interest from the SSQ and IRI questionnaires. Correlations between all the subscales were obtained using Pearson correlations and FDR was used to account for multiple comparisons. Reliability measures were calculated using Cronbach alpha.

### IBS

For the fNIRS IBS analysis, we used general linear mixed models (GLMM), as IBS data derived with WTC follows a beta distribution (ranging from 0 to 1), and these models also account for missing data and nesting. We used the glmmTMB package^48^ to run the GLMM analysis and the *emmeans* package^49^ for *post hoc* analysis (pairwise comparisons). The GLMM model (model 2 specified in the Supplementary material) included valence, social condition, and ROI as main and interacting effects. Dyad ID was added as a random intercept, and random slopes were added for valence, social condition, and ROI. The interaction between random slope and intercept was removed. We also ran individual GLMM models (model 3 specified in the Supplementary material) for each ROI as additional exploratory analyses, with the correlation between random intercept (dyad ID) and random slope removed to allow model convergence across the different GLMM models (these results can be found in the Supplementary material). The FDR method was used to correct multiple comparisons for the number of ROIs. Multiple comparisons in *post-hoc* analyses were also corrected using the FDR method.

### IBS and behavioural/psychological measures

The association between IBS and behavioural/psychological measures was only tested in the *with each other* social condition, as this was the only condition during which significant variation in IBS was observed (see below). To do so, we used four different GLMM models. Each model had valence and ROI as main and interacting effects, and random slopes for valence and ROI. However, the correlation between random intercept (dyad ID) and random slope (valence and ROI) was removed to allow model convergence across the different GLMM models. For the behavioural measures we created a GLMM model to include each dyadic behavioural measure: dyadic average ratings and differences in mother-child average ratings. We added the z-transformed value of the dyadic average ratings and the absolute value of the difference between the mothers’ and children’s subjective valence ratings as a main and interacting effect to their respective models, as well as a random slope. Concerning the association between IBS and psychological measures, we created one GLMM model for each scale (IRI and SSQ) where the z-transformed values of each subscale were added to their respective models as main effects and as interacting effects with valence, social condition and both valence and social condition. All GLMM models for behaviour and psychological measures are specified in the supplementary material (models 4 to 7). The *emtrends* function of the *emmeans* package was used to obtain correlations (and contrasts between correlations) between IBS and behavioural/psychological measures. *P* values were adjusted using FDR, and confidence intervals (CIs) for the contrasts between slopes were corrected using the Bonferroni method (3 estimates).

## Results

### Subjective valence ratings

**Fig. 4:**
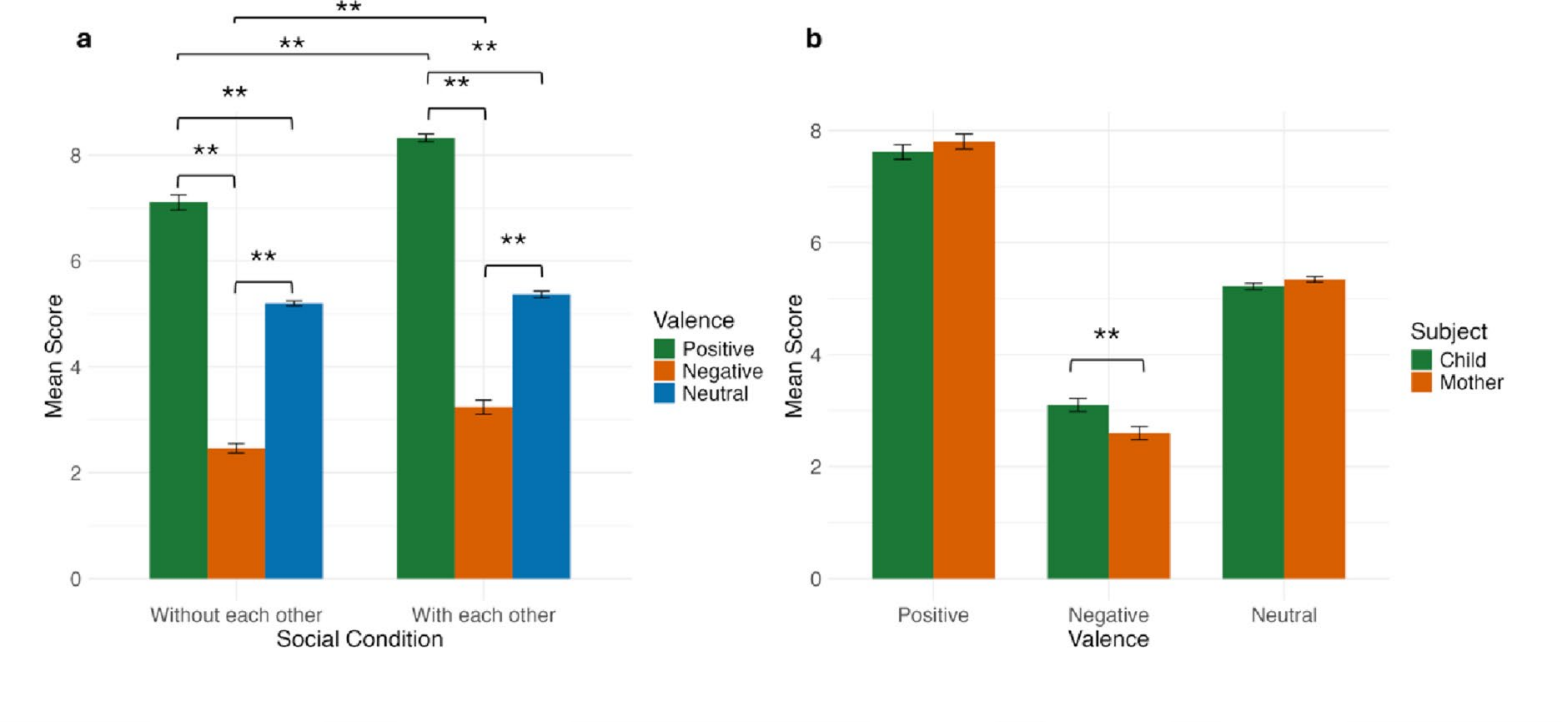
Differences in subjective valence ratings for mothers and children were tested. A mixed model ANOVA showed: (**a**) a significant effect for the interaction between valence - positive, negative, and neutral - and imagined social condition - with or without each other; (**b**) a significant interaction between valence and subject - mother or child (**b**). ** Indicates significant differences found with pairwise *post-hoc* comparisons (*p* < 0.001).

The ANOVA (mixed effects model) results showed significant main effects of valence (*F(*2, 370) = 1134.824, *p* < 0.001) and social condition (*F(*2, 370) = 118.921, *p* < 0.001), of the interaction between valence and social condition (*F(*2, 370) = 24.940, *p* < 0.001) and between valence and subject (*F(*2, 370) = 13.547, *p* < 0.001), no other effects were significant (Supplementary Table S2). To decompose the interaction between valence and social condition, we performed *post hoc* pairwise comparisons. When isolating the effect of valence at each level of social condition, the situations presented to participants were perceived as we intended, with positive situations (M_*without each other*_ = 7.103, SD = 1.241, M_*with each other*_ = 8.321, SD = 0.621) rated significantly higher than neutral (M_*without each other*_ = 5.200, SD = 0.410, M_*with each other*_ = 5.367, SD = 0.523, *p* < 0.001 ) and negative (M_*without each other*_ = 2.463, SD = 0.765, M_*with each other*_ = 3.239, SD = 1.156, *p* < 0.001) situations, and with neutral situations also rated significantly higher than negative ones (*p* < 0.001). When isolating the effect of social condition at each level of valence, positive and negative situations were rated significantly higher in the *with each other* social condition compared to the *without each other* social condition (*p*_positive_ < 0.001, *p*_negative_ < 0.001), i.e., positive situations were perceived as more positive and negative ones as less negative (Fig. 4a and Supplementary Table S3). Regarding the interaction between subject and valence, *post hoc* pairwise comparisons, isolating the effect of subject at each valence level, showed that mothers rated negative situations significantly lower (more negative) than children (M_mother_ = 2.600, SD = 1.032, M_child_ = 3.102, SD = 1.016, *p* = 0.001), while no significant differences were found for positive (M_mother_ = 7.806, SD = 1.160, M_child_= 7.618, SD = 1.147, *p* = 0.177), and neutral situations (M_mother_ = 5.343, SD = 0.441, M_child_ = 5.220, SD = 0.505, *p* = 0.177) (Supplementary Table S4, Fig. 4b).

### Individual difference measures

Descriptive statistics for mothers’ empathy traits from the PT, PD, and EC subscales of the IRI scale, as well as for children’s attachment from the SBS and SHS of the SQQ questionnaire, are summarised in Supplementary Table S5. Only the two subscales of SSQ (SBS and SHS) were significantly correlated (*r* = 0.593, *p* = 0.001; Supplementary Table S6).

### IBS

**Fig. 5:**
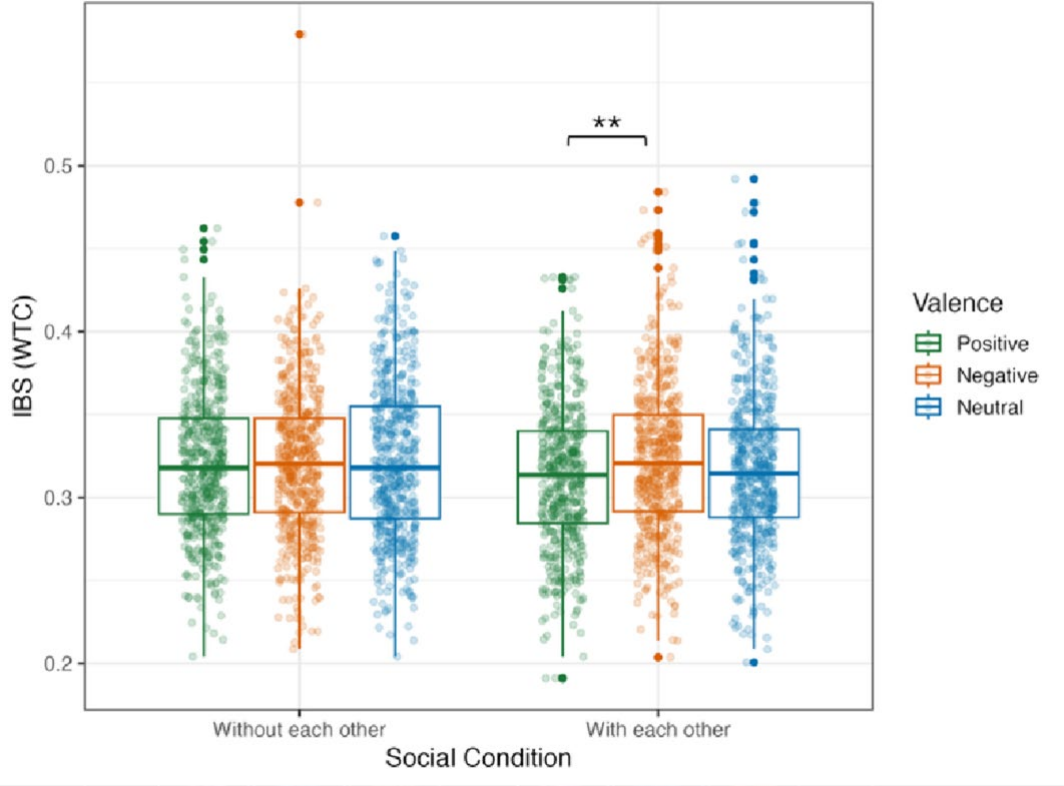
IBS variation according to social condition and valence, across all ROIs. The GLMM model used valence, social condition and ROI as main and interacting factors, and results showed a significant interaction between valence and social condition, with higher IBS for negative situations than positive ones in the with each other condition. IBS values were derived using WTC. Box plot show the median and interquartile range for each valence in each social condition. Dots represent individual data points. **Indicate significant effects after pairwise comparisons of estimated marginal means (*p* < 0.05).

IBS variation was tested by computing a GLMM model with valence, social condition, and ROI as main and interacting effects. We found a main effect of valence (*X*^*2*^ (2) = 6.564, *p* = 0.038) and a significant interaction between valence and social condition (*X*^*2*^ (2) = 7.337, *p* = 0.026). All other effects remained non-significant (Supplementary Table S8). To decompose the significant interaction between valence and social condition, *post hoc* analyses were computed isolating social condition, which revealed no significant differences. When isolating valence, we found a significant difference in the *with each other* social condition (*p* = 0.016) between positive (*emmeans* = 0.313, *SE* = 0.003) and negative (*emmeans* = 0.323, *SE* = 0.003) scenarios, where IBS was higher during negative than during positive situations (Fig. 5 and Supplementary Table S9). This IBS analysis was performed with only the right-handed population, with results supporting the findings from the whole population (Supplementary Tables S24 and S25) .

### Association between IBS and behavioural and psychological measures

We investigated possible relationships between IBS and behavioural and psychological measures within the *with each other* social condition (where a variation in IBS was identified).

#### Association between IBS and behaviour (experienced valence ratings)

The association between behaviour and IBS in the *with each other* social condition was tested with two GLMM models—one for differences in mother-child subjective ratings and one for dyadic average scores— including valence, ROI, and, where appropriate, z-scores from the absolute value of the differences in mother-child subjective ratings and dyadic average scores as main and interacting effects.

**Fig. 6:**
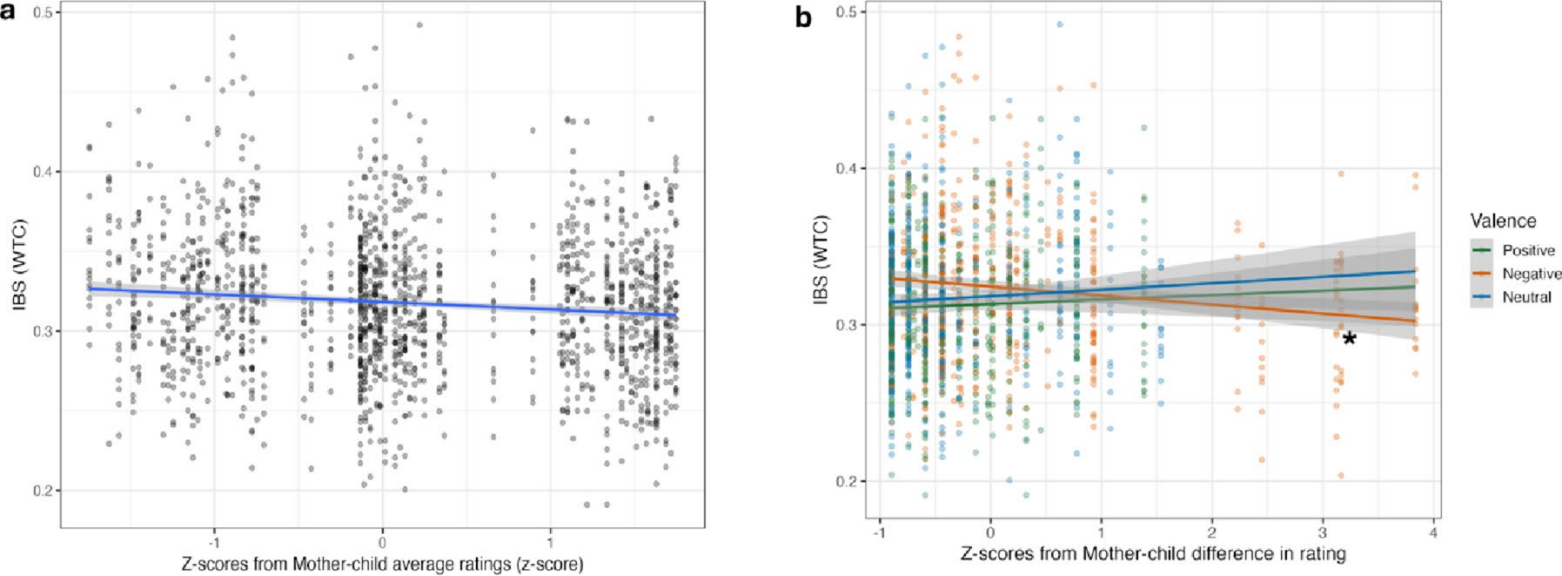
(**a**) Association between IBS and z-scores from dyadic average subjective valence rating scores (SAM scale). The GLMM model revealed a significant main effect of dyadic average scores. The x-axis represents the z-scores from the average dyadic average valence ratings, lower values indicate lower positivity. A lower valence rating is associated with higher IBS. (**b**) Association between IBS and z-scores of the mother-child absolute differences in subjective valence ratings (SAM scale). The GLMM model had valence, ROI and the z-scores of mother-child differences in ratings and showed a significant interaction between valence and mother-child differences in subjective valence ratings. The x-axis represents the z-scores from the differences between mother and child average ratings for each valence, where negative values represent a smaller difference between mother and child subjective valence ratings. Dots represent individual data points. * Indicates significant effects after pairwise comparison of marginal slopes (*emtrends*) ( *p < 0.05 ).

The model focused on dyadic average scores revealed a main effect dyadic average scores (*X*^*2*^(1) = 4.571, *p* = 0.033 ). All other effects remained non-significant (Supplementary Table S12). *Post hoc* analyses revealed a negative correlation between IBS and dyadic average scores (*trend* = -0. 033, *SE* = 0. 031, 95% CI = [-0. 094 0. 027]), suggesting that there was a tendency for IBS to be lower in situations imagined *with each other* rated as more positive by the dyad, independently of valence (Fig. 6a).

The model concerning differences in mother-child scores revealed a main effect of valence (*X*^*2*^ (2) = 12.550, *p* = 0.002) and a significant interaction between differences in subjective ratings and valence (*X*^*2*^ (2) = 6.562, *p* = 0.038). All other tested effects remained non-significant (Supplementary Table S13). To decompose the significant interaction between differences in subjective ratings and valence, *post hoc* analyses were computed by isolating the factor valence. These showed a significant negative correlation between IBS and differences in mother-child subjective ratings for negative situations (*trend* = -0.024, *SE* = 0.010, 95% CI = [-0.043 -0.004], *p* = 0.016) (Fig. 6b). No other correlations or the contrast between correlations were significant (Supplementary Table S14 and Table S15). These findings showed that the more similar mothers’ and children’s negative valence ratings were, the higher was IBS in negative situations imagined *with each other*.

#### Associations between IBS and individual difference measures – mothers’ empathy traits

**Fig. 7:**
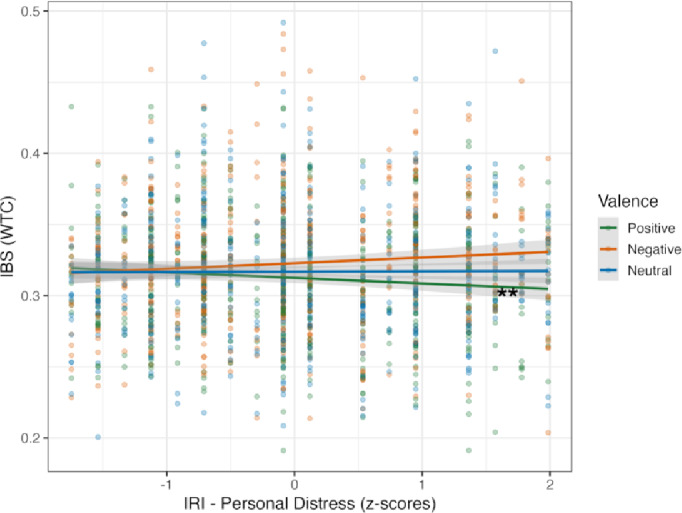
Association between IBS and mother’s personal distress (PD) according to valence. The GLMM model included valence, ROI and z-scores from the PD subscale, and showed a significant interaction between valence and PD. The x-axis represents the z-scores from the Personal Distress dimension of IRI, where greater scores represent a higher tendency to feel distressed by others’ discomfort. Dots represent individual data points. ** Indicates significant effects after pairwise comparison of marginal slopes (*emtrends*) (*p* < 0.05).

The association between mothers’ empathy (IRI) and IBS during situations imagined *with each other* (*N* = 38) was analysed by running a GLMM model using valence, ROI and the z-scores from the IRI subscales of PD, PT, and EC as factors. The GLMM model revealed a significant main effect of valence (*X*^*2*^ (2) = 10.713, *p* = 0.005) and of the interaction between valence and PD (*X*^*2*^ (2) 6.150, *p* = 0.046), with no other significant effects (Supplementary Table S18). *Post-hoc* analyses showed that IBS was significantly negatively correlated with PD in the positive valence (*trend* = -0.020, *SE* = 0.009, CI [-0.038 -0.002], *p* = 0.032), and that there was a significant contrast between positive and negative valences (*estimate* = -0.036, *SE* = 0.015, CI [-0.072 -0.001], *p* = 0.049) (Fig. 7). No other correlations or contrasts were significant (Supplementary Table S19 and Table S20). These findings showed that dyads in which mothers scored higher on PD displayed lower IBS in imagined positive scenarios, with the opposite pattern observed in negative situations.

#### Associations between IBS and individual difference measures – children’s attachment security

**Fig. 8:**
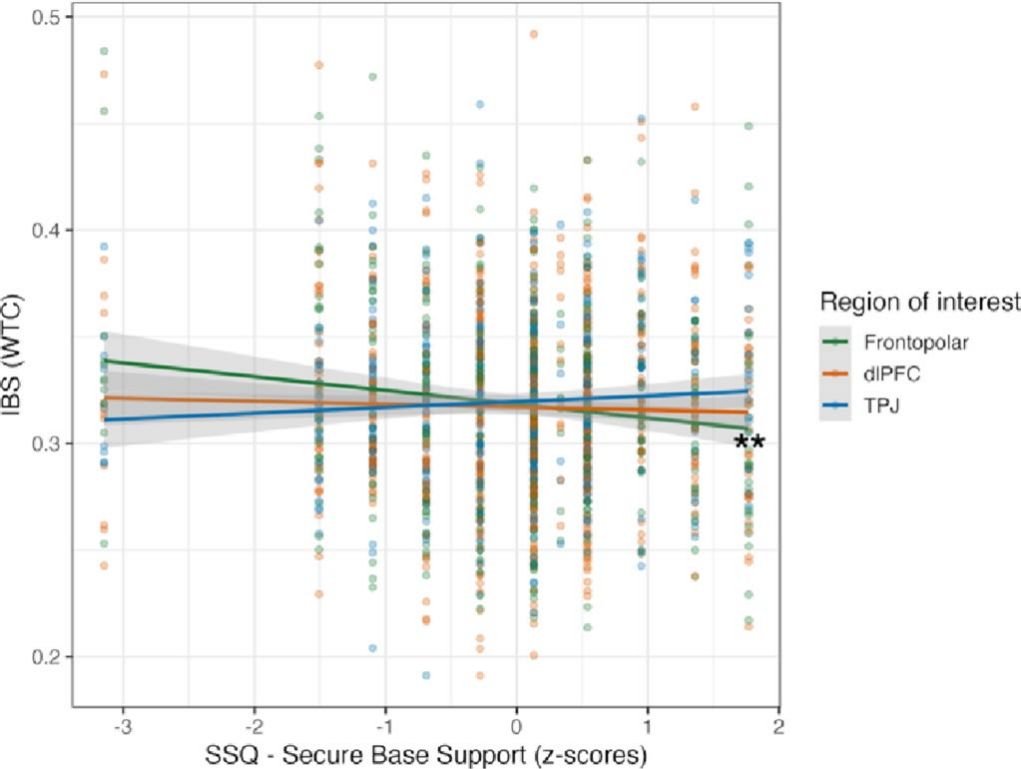
Association between mother-child IBS and child’s attachment security to the mother, according to ROI. The GLMM model included valence, ROI and z-scores from Secure Base Support (SBS) subscale, and showed a significant interaction between ROI and SBS. The x-axis represents the z-scores from SBS; higher scores represent higher attachment security to the mother. Dots represent individual data points. ** Indicates significant effects after Pairwise comparison of marginal slopes *p* < 0.05).

The association between children’s attachment to their mothers and mother-child IBS during situations imagined *with each other* (*N* = 36) was analysed by adding the SHS and SBS subscales of the SSQ, as well as valence and ROI as main and interaction effects to the GLMM model. The model revealed a main effect of valence (*X*^*2*^ (2) = 9.095, *p* = 0.011) and a significant interaction effect between ROI and SBS (*X*^*2*^ (2) = 6.416, *p* = 0.040). No other significant results were observed (Supplementary Table S21). *Post-hoc* analyses showed a significant negative correlation between SBS scores and IBS in the right frontopolar area (*trend* = -0.029, *SE* = 0.013, CI [-0.054 -0.003], *p* = 0.026), and a significant contrast between frontopolar and TPJ areas (*estimate* = -0.041, *SE* = 0.017, CI [-0.082 -0.001], *p* = 0.040) (Fig. 8). No other *trends* or contrasts were significant (Supplementary Table S22 and Table S23). These findings showed that dyads in which children reported lower attachment security to their mother displayed increased IBS in the right frontopolar area across all valences of the imagined *with each other* scenarios.

## Discussion

This study investigates the hypothesis that IBS in mother-child dyads varies during *imagined* emotional experiences of different valences (positive, negative and neutral) and social conditions (*with* and *without* the mother’s presence). Previous research on IBS using co-presence paradigms has focused on the potential effects of *external* emotional stimuli^11^ and *physical* co-presence^13^. However, the possible influence of mothers’ and pre-adolescents’ own, *internally generated* emotional experiences, as well as the mother’s *imagined* presence, has not yet been explored. In our study, IBS varied across emotional valences only in the *with each other* condition, where IBS was higher during imagined negative scenarios than positive ones. Furthermore, dyads in which mothers scored higher on personal distress (PD) exhibited higher IBS in negative scenarios but lower IBS in positive ones imagined *with each other*. Additionally, dyads in which children reported lower secure base support (SBS) related to the mother displayed increased IBS in the right frontopolar area across all valences imagined *with each other*.

When children and their mothers imagined being together in positive and negative situations targeting the child, their individual subjective valence ratings showed that they felt better – i.e., they rated positive situations as more positive and negative situations as less negative (though still negative) – compared to imagining being *without each other*. This finding suggests that, even in the absence of explicit task instructions to engage in emotion regulation (ER)^50^– i.e., only to pay attention to subjective emotions –, the mere imagined presence of the mother was associated with a change in subjective emotion experience towards less negativity.

For children, feeling better when they imagined being with their mothers may have reflected a subjective sense of security derived from their mother’s presence. Our findings align with previous works reporting that pre-adolescents still rely on their parents for emotional support, feeling better in their presence—regardless of the emotional valence of the situation^20,22^— and that the availability of social support positively influences children (reviewed in ref.^51,52^. Furthermore, reduced negative emotion in children during negative situations imagined with their mothers present might reflect a decrease in the energetic costs associated with ER, as proposed by Social Baseline Theory (SBT). SBT suggests that individuals perceive others as extensions of the self and, in times of distress, tend to seek social support to enable co-regulation by making use of others’ bioenergetic resources, which is thought to reduce the energetic costs of ER^53,54^.

Mothers also reported feeling better when they imagined being together with their children. This finding may reflect the rewarding experience of maternal caregiving. This is aligned with previous research showing that parents report increased happiness when spending time with their children^55^, which is linked with increased brain activation in reward and motivation networks in response to infants’ cues^56–59^. From another perspective, studies showed that mothers reported experiencing less guilt when being present with their child^60^. Therefore, feeling better when imagining being with their children might also reflect a reduction in maternal guilt. Furthermore, helping others is often rewarding for the helper (reviewed in ref.^61^, which is visible, for example, in compassion acts. Compassion involves concern about others’ well-being and motivation to alleviate their suffering^28^. Compassion displays are associated with increased activity in brain areas related to reward and pro-social behaviours (reviewed in ref.^62^, further supporting the hypothesis that helping others may have a positive effect on the helper. Imagining being able to be present for their children might reduce mothers’ negative feelings due to the rewarding nature of compassion in the context of caregiving. Overall, our findings extend the existing literature by showing that being present when children face positive and negative situations contributes to mothers feeling better.

IBS varied according to valence in scenarios where mothers and children imagined being together *versus* being apart. The effect of *physical* presence on IBS was documented previously. For example, IBS was observed to increase when adult participants listened to positive and neutral stimuli together in the same room as compared to being alone in separate rooms^13^. Similarly, another study reported that IBS was higher when university students completed math tasks next to each other, without visual contact, compared to performing the same task in separate rooms^14^. Our findings extend these results by showing that in mother-child dyads, the *imagined* presence *versus* absence of the mother during emotional situations of different valences targeting the child is sufficient to have a modulating effect on IBS.

Imagining being *with each other* in emotional situations targeting the child led to an overall modulation of IBS across all three ROIs in mother-child dyads. Specifically, during the *with each other* social condition, we observed a significant decrease in overall IBS during positive situations as compared to negative ones.

Our findings of a negative *versus* positive IBS difference across all ROIs are consistent with previous findings focusing on IBS differences as a function of stimulus valence, where IBS was observed to generally be higher in response to negative (*versus* neutral and/or positive) stimuli. For example, in a sequential fMRI study, adult participants showed higher IBS when viewing film clips with more negative valence, in regions involved in emotional processing and the default mode network, including the TPJ^63^. Using the same methodology, participants showed higher IBS when narrating/listening to emotional episodes of more negative valence (*versus* more positive) in areas including the rTPJ, dlPFC or superior parietal lobe, for example^64^. Our study extends this knowledge by having participants experience subjective emotions associated with imagined scenarios rather than reacting to external stimuli, suggesting a modulating role of both valence and social context on IBS within this new framework.

Why might IBS have been relatively decreased and thus children’s and mothers’ brain activities less similar during positive than negative situations imagined *with each other*? One possible explanation may be offered by behavioural valence ratings. On the one hand, when testing the association between IBS and dyadic average valence ratings, there was a main effect of valence ratings. Specifically, there was a negative correlation between IBS during the *with each other* condition and dyadic average valence ratings—indicating that the more positively dyads perceived the imagined situations on average across both mothers and children, the lower was their IBS. On the other hand, we looked at differences between mothers’ and children’s valence ratings and their association with IBS. This analysis revealed a significant interaction between IBS during the *with each other* condition and differences between mothers’ and children’s valence ratings–i.e., a negative correlation between IBS and specifically differences between mothers’ and children’s valence ratings for negative scenarios. In other words, the more similarly mothers and children rated negative scenarios during the *with each other* condition, the higher their IBS. Furthermore, the negative valence was also the one for which higher dissimilarity in valence ratings was observed. This might have led to greater variability within the negative valence ratings, contributing to the observed significant negative correlation. Such variability might reflect differences in emotional appraisal between dyad members when processing negative content.

We are aware that the association between IBS and valence ratings is correlational and not causal. Any interpretations of this relation therefore need to be regarded with caution. Nonetheless, our findings suggest that a similar subjective experience of positively and negatively valenced scenarios during the *with each other* condition could–at least partially–explain the difference in IBS. Such an interpretation aligns with a proposed role of IBS in mutual prediction and subjective valence attribution processes^65,66^. In our study, this effect was more pronounced for negative (than positive) scenarios. While speculative, one may refer to the Broaden-and-Build Theory of Positive Emotions^67^ for insights. This theory suggests that positive emotions are associated with a broader range of attentional, thought and behavioural patterns than negative emotions, which are typically linked to more specific behavioural signatures and a reduced scope of attentional, thought and behavioural patterns. Therefore, a higher similarity in how negative emotions were perceived by mother-child dyads in the *with each other* condition may have led to more similar cognitive processing, mutual prediction and subjective valence attribution of negative situations. However, the role of IBS during negative situations still needs to be further explored—it may be associated with empathic processes that allow the observer (here mothers) to better understand children’s emotional state, or indicate that both participants were similarly distressed by imagining being together in negative situations targeting the child (see below).

An open question remains why IBS only showed a differential pattern between positive and negative scenarios during the *with each other* but not in the *without each other* condition. We also exploratively checked associations between IBS during the *without each other* condition and both dyadic average valence ratings and differences between mothers’ and children’s valence ratings, and found no significant associations (Supplementary Tables S16 and S17). It might therefore be that mothers’ and children’s subjective experiences during the *without each other* condition were more dissimilar, which may be reflected in the less differentiated IBS pattern. Conversely, IBS during the *without each other* condition might have been more strongly driven by external information processing, which was similar across valences. A further explanation for why valence-related IBS variations were only observed in the *with each other* social condition may be that being physically present in the same room facilitated the imagination of these scenarios. This hypothesis aligns with previous research showing that, even in the absence of physical touch or visual contact, adult participants who were co-present with their romantic partners in the same room attended to each others’ breathing and reported actively thinking about their partners^68^.

IBS across ROIs was associated with mothers’ empathy traits. Specifically, mothers’ tendency to become distressed by others’ emotions (PD) was the only empathy trait correlated with IBS in situations imagined during the *with each other* social condition, depending on valence. In dyads in which mothers indicated having a stronger tendency to feel PD, mother-child IBS was decreased during positive imagined situations but increased during negative ones, across all ROIs. A tendency to feel PD indicates that an individual is more likely to become emotionally overwhelmed by others’ suffering^33^, and is linked to decreased helping behaviour, as the focus is more on reducing one’s own rather than others’ distress^69^. In our study, dyads with mothers who scored higher on PD might therefore have been more neurally attuned in negative situations imagined *with each other* due to subjectively experiencing more similar feelings, particularly emotional distress. Such an interpretation accords with our findings of associations between valence ratings and IBS, with particularly higher IBS for negative situations imagined *with each other* when mothers’ and children’s valence ratings were more similar.

Such a pattern of findings begs the question of whether more synchrony may always be better. As previously suggested^6^, this may not be the case, especially within stressful situations where high synchrony may fuel the propagation of negative affect across interaction partners^70^. Along the same lines, increased IBS over the dlPFC during mother-child problem-solving was observed in dyads comprising mothers with an insecure (*versus* secure) adult attachment classification, indicating increased attentional processes and regulatory demands and thus a potential compensatory mechanism for otherwise less attuned interaction and relationship elements^16^. More research is therefore needed on the potential benefits *versus* pitfalls of high IBS, depending on the task setting and individual participant characteristics.

Finally, our data also suggests an association between children’s secure base support (SBS) scores and IBS, with dyads in which children reported lower attachment security to their mothers displaying increased IBS in the right frontopolar area across all valences of the scenarios imagined *with each other*.

The frontopolar cortex is a region associated with the processing of one’s own and others’ feelings^71^. According to the above-mentioned considerations of more synchrony not always being better, higher IBS in the frontopolar cortex in dyads with children scoring lower on SBS security may reflect a potential compensatory mechanism for otherwise less attuned interaction and relationship elements that require stronger dyadic attention and/or regulation^16^. While a previous study observed such an association between IBS and attachment with an attachment measure obtained from mothers^16^, our new data shows a similar pattern with an attachment measure obtained from children. Together, these findings warrant further exploration of associations between measures of attachment and IBS during dyadic social interaction^27,72^.

## Implications

The findings from this study suggest that higher IBS, particularly in negative contexts, might not always be beneficial and highlight the importance of individual characteristics in shaping dyadic interactions. While empathy helps us understand and support others when they face challenges, feeling *with* others (personal distress) instead of feeling *for* others (empathic concern) can lead to shared distress and reduce the likelihood of helping^62,69^. In the parent-child context, parents’ capacity to deal with others’ emotions is important, as a tendency to be distressed by others’ suffering can create a need to withdraw, impacting their availability to support their children who rely on them for support. However, when individuals are skilled at ER, personal distress can be regulated, allowing the engagement of empathic concern and compassion^73,74^.

## Limitations and future research

This study explored variations in IBS across valences and social conditions during an imagery task, also concerning individual differences in empathy and attachment. Some limitations to our findings may apply.

Our results on children’s attachment measures should be interpreted with caution, as the Cronbach’s alpha for the SBS subscale was somewhat low. This may reflect a relatively small sample size (in which concerns behavioural measures) and the limited number of items in this subscale. Additionally, while our sample falls within the scale’s validated age range (10–14 years), its higher mean age compared to the original validation study^35^ could contribute to variations in item interpretation.

Our focus in this study was on the effect of the imagined presence *versus* absence of another person in an emotional context, when participants were in the same room. Future studies could explore how physical presence influences this effect by conducting the study with participants in separate rooms. Additionally, our sample was mainly composed of mothers with a higher education degree. In future studies, it would also be interesting to focus on populations with lower education status, as education has been observed to be associated with variability in brain regions involved in mentalizing processes^75^.

## Conclusion

Recent studies implicate IBS in dyadic processing of emotional experiences during physical presence studies. Our findings extend that knowledge by showing that IBS varies depending on the social context and the valence of the information being processed in imagined situations. Specifically, mother-child dyads showed higher IBS in negative situations than positive ones imagined together, suggesting that each other’s presence is experienced differently depending on valence. Furthermore, IBS during emotional experiences seems to be associated with mothers’ empathy and children’s attachment. Overall, our findings extend previous knowledge by showing that the imagined presence of a partner is related to IBS variations in a valence and trait-dependent manner.

## Supplementary Information

Below is the link to the electronic supplementary material.

Supplementary Material 1

## Data Availability

The datasets generated during and/or analysed during the current study are available from the corresponding author on reasonable request.
