# Supplementary material for: Modulation of interbrain synchrony by emotional valence and maternal presence in mother–child dyads: neural links to empathy and attachment
Source: Sci Rep. 2026 Mar 16;16:13692. doi: 10.1038/s41598-026-43086-7 (PMC13125508; doi:10.1038/s41598-026-43086-7)
Supplement: Supplementary file 1 — Supplementary Material 1 [file 41598_2026_43086_MOESM1_ESM.docx]

# Supplementary material

**S1. Electrodermal activity**

### **Set-up and data collection parameters**

Electrodermal activity (EDA) data were simultaneously acquired from both participants using a Biopac MP150 setup with two GSR100C modules (one for mothers and one for children). Disposable electrodes were attached between the digital and medial phalanges of each participant’s index and middle fingers (non-dominant hand) using electrode gel (Gel101 from Biopac) before starting the data acquisition. EDA was collected at 1000 Hz, with low (10 Hz) and high-pass filtering to remove high-frequency noise and the DC (Direct Current) component using the Acqknowledge 5.0 software (Biopac Systems, Inc., USA).

### **EDA Analysis and data quality assessment**

Data was exported from Acqknowledge in *.mat* format and all analyses were then conducted in MATLAB (2024b) using the MATLAB-based software Ledalab (V3.4.9). RAW files were converted to the Ledalab format and pre-processed using a moving average filter to remove motion artefacts. Ledalab batch mode was subsequently used to down-sample the data to 1 Hz and to analyse it using Continuous Decomposition Analysis (CDA) – which decomposes the EDA signal into phasic and tonic components (Benedek & Kaernbach, 2010). The CDA analysis was used to derive phasic information from EDA, with the event-related response window set to 1 - 12 s after stimulus onset (corresponding to the imagery period) after stimuli onset, and the minimum threshold for EDA peak detection set to 0.01 muS.

The pre-processed data were then visually inspected for quality by plotting the skin conductance values. Runs with too much noise were excluded from the analysis and events occurring during movement spikes were also removed from the final data. Participants’ data were excluded if they had less than four imagery periods per valence for each social condition with good EDA data quality. After data quality assessment, we included EDA data from 34 children and 32 mothers. Two participants only had data from one run in each condition (*with each other* versus *without each other*) from the fNIRS data, so only the data from those runs was taken from EDA as well.

### **Arousal as measured by EDA**

Arousal levels were derived from the skin conductance response (SCR) by using the average phasic driver within the response window variable (CDA.SCR in Ledalab output) for all imagery periods. Phasic driver values were then averaged across all imagery periods of the same valence and social condition, yielding six SCR values per participant (3 valences x 2 social conditions).

For data analysis, the data was normalised by subtracting the average phasic driver (CDA.SCR) of the 8 s preceding each imagery period (corresponding to the fixation cross) from the same SCR metric of the respective imagery period. To calculate the average phasic driver values for the fixation cross, the CDA analysis parameters were changed to match the 1 to 8s response window. This normalization was done to allow comparisons between participants.

**S2. Statistical analyses**

Specified below are the statistical models for behavioural and IBS measures, as well as for the association between IBS and behaviour and IBS and psychological measures, and EDA. After accounting for missing data, the ratios between the number of data points and the number of estimated parameters for the models were higher than 30.

**Behaviour:**

1. ART model: *Valence rating ~ Valence * Social Condition * Subject + (1|Participant ID)*

**IBS:**

1. Full GLMM model: *IBS ~ Social Condition * Valence * ROI +*

*(1+ Social Condition + Valence + ROI|| Dyad)*

1. Individual GLMM models: *IBS ~ Social Condition * Valence +*

*(1 + Social Condition + Valence || Dyad)*

**IBS * behaviour/psychological measures:**

1. Dyadic average valence scores GLMM: *IBS ~ Valence * ROI * Dyadic average scores + (1 + Valence + ROI + Dyadic average scores|| Dyad)*
2. Differences in valence scores GLMM*: IBS ~ Valence * ROI * Differences in scores +*

*(1 + Valence + ROI + Differences in scores|| Dyad)*

1. IRI GLMM: *IBS ~ Valence + IRI_PD + IRI_PT + IRI_EC + ROI + Valence:IRI_PD + Valence:IRI_EC + Valence:IRI_PT + ROI:IRI_PD + ROI:IRI_EC + ROI:IRI_PT + ROI:Valence:IRI_PD + ROI:Valence:IRI_EC + ROI:Valence:IRI_PT*

*+ (1 + Valence + ROI || Dyad)*

1. SSQ GLMM*: IBS ~ Valence + SSQ_SBS + SSQ_SHS + ROI + ROI:Valence + ROI:SSQ_SBS + ROI:SSQ_SHS + Valence:SSQ_SBS + Valence:SSQ_SHS + ROI:Valence:SSQ_SBS + ROI:Valence:SSQ_SHS*

*+ (1 + Valence + ROI || Dyad)*

**EDA:**

1. ART model: *Mean score ~ Valence * Social Condition + (1|Participant)*

**S3. Arousal as measured by EDA results**

To explore arousal derived from EDA, we calculated a mixed-effects ANOVA. Results (Table S7) revealed similar arousal over all conditions tested. There was no main effect of valence (*F*(2, 325) = 1.051,  *p* = 0.351) or social condition (*F*(1, 325) = 1.577, *p* = 0.210), and no significant interaction between valence and social condition (*F*(2, 325) = 1.900, *p* = 0.151).

### **S4. Exploratory analyses: IBS in each ROI**

While our main analyses showed a significant valence by social content interaction across all ROIs, they did not reveal any interaction between valence, social condition and ROI. Nonetheless, we exploratorily looked at the valence by social condition interaction within each ROI separately by correcting for the number of ROIs with the FDR method. When doing so, we only found a significant valence by social condition interaction in the right dlPFC (*X*^2^(2) = 12.3398, *p*_corrected_ = 0.006) (see Table S10 for a summary of the GLMM results for all three ROIs). In accordance to what was found in the analyses across all ROIs, *post hoc* analyses showed that in the *with each other* social condition, IBS was significantly higher in negative (*emmeans* = 0.324, *SE* = 0.003) compared to positive (*emmeans* = 0.321, *SE =* 0.003) imagined situations (*p* = 0.032; Figure S3; Table S10), after FDR correction. IBS patterns for the frontopolar area and TPJ can be found in the Supplementary Figure S2 and Figure S3, respectively.

A significant negative > positive IBS difference during the with each other condition was observed only in the right dlPFC, a region involved in attention and regulatory processes, including emotion regulation^1–3^. The dlPFC has previously been implicated in IBS studies focusing not only on parent-child cooperation^4^ but also on studies comprising various emotional tasks. For example, Wang and colleagues (2022) observed an increase in IBS in the right dlPFC (besides in the TPJ) when participants shared negative emotional memories with listeners (here university students with psychology teachers), compared to reading numbers^5^. Another study looked at IBS during a social exclusion task involving adult dyads, with one dyad member being the target of social exclusion and the other an observer. The results showed that IBS in the right dlPFC was higher during social exclusion^6^. These studies support our finding that the right dlPFC plays a role in the dyadic processing of emotions associated with regulation and attention processes, particularly during negative situations.

### **S5. Exploratory analyses: IBS variation in the right-handed population**

We exploratorily conducted our main IBS analyses only with the right-handed dyads, to test it with a more uniform lateralisation. Our results were unchanged from the results reported for the entire sample. However, the difference in IBS between social conditions for the positive valence was significant (p=0.047, see Table S24 and S25).

### **S6. Exploratory analyses: Correlation between arousal and differences in mother-child valence ratings for negative scenarios**

A possible interpretation for a seemingly stronger IBS in the dlPFC and frontopolar areas during negative scenarios is that anxiety during these scenarios was higher and/or the need for emotion regulation is shared by mother and child. To test this hypothesis, we exploratorily tested the correlation between mother-child absolute differences in valence ratings during the negative scenarios and mother-child average arousal, mothers’ arousal and children’s arousal. Results showed no significant correlations (see Table S26).

**Supplementary tables**

*Table S1Channels’ main specificities according to the fOLD toolbox and ROI distribution.*

| Channel | Area | Specificity (%) | Area | Specificity (%) | ROI |  |
| --- | --- | --- | --- | --- | --- | --- |
| 1 | Frontopolar Area (BA 10) | 54 | Orbitofrontal Area (BA 11) | 45 | Frontopolar |  |
|  |  |  |  |  |  |  |
| 2 | Frontopolar Area (BA 10) | 87 | Dorsolateral Prefrontal Cortex (BA 9) | 5 | Frontopolar |  |
|  |  |  |  |  |  |  |
| 3 | Frontopolar Area (BA 10) | 69 | Orbitofrontal Area (BA 11) | 22 | Frontopolar |  |
|  |  |  |  |  |  |  |
| 4 | Frontopolar Area (BA 10) | 72 | Dorsolateral Prefrontal Cortex (BA 9) | 17 | Frontopolar |  |
|  |  |  |  |  |  |  |
| 5 | Dorsolateral Prefrontal Cortex (BA 9) | 52 | Dorsolateral Prefrontal Cortex (BA 46) | 26 | dlPFC |  |
|  |  |  |  |  |  |  |
| 6 | Dorsolateral Prefrontal Cortex (BA 46) | 49 | Pars Triangularis Broca's (BA 45) | 32 | dlPFC |  |
|  |  |  |  |  |  |  |
| 7 | Frontopolar Area (BA 10) | 31 | Orbitofrontal Area (BA 11) | 30 | Frontopolar |  |
|  |  |  |  |  |  |  |
| 8 | Pars Triangularis Broca's (BA 45) | 44 | Dorsolateral Prefrontal Cortex (BA 46) | 43 | dlPFC |  |
|  |  |  |  |  |  |  |
| 9 | Dorsolateral Prefrontal Cortex (BA 9) | 62 | Frontopolar Area (BA 10) | 20 | dlPFC |  |
|  |  |  |  |  |  |  |
| 10 | Dorsolateral Prefrontal Cortex (BA 9) | 69 | Includes Frontal Eye Fields (BA 8) | 29 | dlPFC |  |
|  |  |  |  |  |  |  |
| 11 | Dorsolateral Prefrontal Cortex (BA 9) | 69 | Dorsolateral Prefrontal Cortex (BA 46) | 29 | dlPFC |  |
|  |  |  |  |  |  |  |
| 12 | Dorsolateral Prefrontal Cortex (BA 9) | 62 | Dorsolateral Prefrontal Cortex (BA 46) | 26 | dlPFC |  |
|  |  |  |  |  |  |  |
| 13 | Includes Frontal Eye Fields (BA 8) | 58 | Dorsolateral Prefrontal Cortex (BA 9) | 33 | dlPFC |  |
|  |  |  |  |  |  |  |
| 14 | Superior Temporal Gyrus (BA 22) | 35 | Middle Temporal Gyrus (BA 21) | 35 | TPJ |  |
|  |  |  |  |  |  |  |
| 15 | Superior Temporal Gyrus (BA 22) | 36 | Retrosubicular area (BA 48) | 19 | TPJ |  |
|  |  |  |  |  |  |  |
| 16 | Angular Gyrus (BA 39) | 35 | Superior Temporal Gyrus (BA 22) | 26 | TPJ |  |
|  |  |  |  |  |  |  |
| 17 | Angular Gyrus (BA 39) | 53 | Supramarginal Gyrus (BA 40) | 30 | TPJ |  |
|  |  |  |  |  |  |  |
| 18 | Angular Gyrus (BA 39) | 80 | V3 (BA 19) | 17 | TPJ |  |

*Table S2 Results from the ANOVA analyses for individual subjective valence ratings with valence, social condition and subject as fixed and interacting effects. A significant Effect was observed for valence, social condition, the interaction between valence and social condition and between valence and subject.*

| Effect | F | Df | Df. Residual | *p-value* |
| --- | --- | --- | --- | --- |
| Valence | 1134.824 | 2 | 370 | <0.001 |
| Social Condition | 118.921 | 1 | 370 | <0.001 |
| Subject | 1.306 | 1 | 74 | 0.257 |
| Valence*Social Condition | 24.940 | 2 | 370 | <0.001 |
| Valence*Subject | 13.547 | 2 | 370 | <0.001 |
| Social Condition*Subject | 1.886 | 1 | 370 | 0.171 |
| Valence*Social Condition*Subject | 0.410 | 2 | 370 | 0.664 |

*Table S3 Post hoc contrasts between subjective valence ratings across valences and social conditions, based on the results from Table S2. All contrasts were significant, except for the contrast between social conditions concerning the neutral valence. Ng = Negative, Pos = Positive, Nt = Neutral. P values corrected with the FDR method.*

|  | Contrast | SE | df | t-value | p-value |
| --- | --- | --- | --- | --- | --- |
| Without each other | Ng– Pos | 8.87 | 370 | -31.505 | <0.001 |
|  | Ng– Nt | 8.87 | 370 | -18.219 | <0.001 |
|  | Nt– Pos | 8.87 | 370 | -13.285 | <0.001 |
| With each other | Ng– Pos | 8.87 | 370 | -33.358 | <0.001 |
|  | Ng– Nt | 8.87 | 370 | -14.219 | <0.001 |
|  | Nt– Pos | 8.87 | 370 | -19.139 | <0.001 |
| Negative | without each other – with each other | 8.87 | 370 | 5.670 | <0.001 |
| Positive | without each other – with each other | 8.87 | 370 | 7.523 | <0.001 |
| Neutral | without each other – with each other | 8.87 | 370 | 1.669 | 0.096 |

*Table S4: Post hoc contrasts between subjective valence ratings across valences and subject, based on the results from Table S2. A significant difference between mother and child was observed in the negative valence. P values corrected with the FDR method.*

|  | Contrast | SE | df | t-value | p-value |
| --- | --- | --- | --- | --- | --- |
| Positive | Mother-Child | 8.69 | 359 | -1.352 | 0.177 |
| Negative |  | 8.69 | 359 | 3.619 | 0.001 |
| Neutral |  | 8.69 | 359 | -1.459 | 0.177 |

*Table S5 Means, standard deviations, range, and Cronbach alpha for IRI and SSQ subscale scores.*

| Subscale | Mean | S.D. | Min.-Max. | Cronbach alpha |
| --- | --- | --- | --- | --- |
| IRI - PD | 1.594 | 0.801 | 0.017 – 3.167 | 0.816 |
| IRI – PT | 2.973 | 0.476 | 2.000 – 4.000 | 0.471 |
| IRI – EC | 3.360 | 0.447 | 2.333 – 4.000 | 0.544 |
| SSQ - SBS | 3.383 | 0.349 | 2.286 – 4.000 | 0.494 |
| SSQ - SHS | 3.378 | 0.377 | 2.500 – 3.929 | 0.728 |

*Table S6 Pearson correlations between subscales from IRI and SSQ. **p<0.01*

|  | IRI – PT | IRI – PD | IRI -EC | SSQ – SBS | SSQ - SHS |
| --- | --- | --- | --- | --- | --- |
| IRI – PT | - | 0.113 | 0.247 | -0.119 | -0.130 |
| IRI – PD | - | - | 0.261 | 0.024 | -0.113 |
| IRI – EC | - | - | - | 0.008 | -0.118 |
| SSQ – SBS | - | - | - | - | 0.593** |

*Table S7 Mean and standard deviation for arousal (compared to the baseline) for both mother (n = 32) and child (n = 34), according to social condition and valence. Units in muS, (S.D.)*

| Without each other | | | With each other | | |
| --- | --- | --- | --- | --- | --- |
| Positive | Negative | Neutral | Positive | Negative | Neutral |
| -0.048 (0.138) | -0.013 (0.103) | -0.066 (0.125) | -0.047 (0.161) | -0.004 (0.175) | -0.023 (0.208) |

*Table S8 Results from the GLMM model including valence, social condition and ROI as fixed and interacting factors. Results showed a significant effect of valence and a significant interaction between valence and social condition.*

| Effect | *X*^2^ | | Df | *p-value* | |
| --- | --- | --- | --- | --- | --- |
| Social Condition | 2.819 | 1 | | | 0.093 |
| Valence | 6.564 | 2 | | | **0.038** |
| ROI | 3.042 | 2 | | | 0.219 |
| Social Condition * Valence | 7.337 | 2 | | | **0.026** |
| Social Condition * ROI | 2.235 | 2 | | | 0.327 |
| Valence * ROI | 2.017 | 4 | | | 0.733 |
| Social Condition * Valence * ROI | 6.892 | 4 | | | 0.142 |

*Table S9 Post hoc results for the interaction between valence and social condition across all ROIs, based on the GLMM results from Table S8. P values are FDR corrected. emmeans: 1st value corresponds to the first input in the contrast column.*

|  | Contrast | *emmeans* | SE | p-value |
| --- | --- | --- | --- | --- |
| Without each other | Pos - Ng | 0.320; 0.321 | 0.013 | 0.999 |
|  | Pos – Nt | 0.320; 0.322 | 0.014 | 0.999 |
|  | Ng– Nt | 0.321; 0.322 | 0.015 | 0.999 |
| With each other | Pos - Ng | 0.313; 0.323 | 0.012 | 0.**016** |
|  | Pos – Nt | 0.313; 0.317 | 0.014 | 0.634 |
|  | Ng– Nt | 0.323; 0.317 | 0.015 | 0.535 |
| Negative | without each other – with each other | 0.321; 0.323 | 0.014 | 0.999 |
| Positive | without each other – with each other | 0.320; 313 | 0.015 | 0.067 |
| Neutral | without each other – with each other | 0.322; 0.317 | 0.014 | 0.499 |

*Table S10 GLMM results for each individual ROI after FDR correction. Only the interaction between valence and social condition in the dlPFC remained significant.*

| Effect | dlPFC | | | Frontopolar | | | TPJ | | |
| --- | --- | --- | --- | --- | --- | --- | --- | --- | --- |
|  | *X*^2^ | Df | *p-value* | *X*^2^ | Df | *p-value* | *X*^2^ | Df | *p-value* |
| Social Condition | 2.597 | 1 | 0.161 | 0.035 | 1 | 0.852 | 4.712 | 1 | 0.899 |
| Valence | 2.343 | 1 | 0.465 | 4.322 | 1 | 0.346 | 0.847 | 1 | 0.655 |
| Social Condition * Valence | 12.340 | 2 | 0.006 | 1.339 | 2 | 0.768 | 0.177 | 2 | 0.915 |

*Table 11 Post-hoc results for the valence and social condition interaction in the rdlPFC, after Tuckey correction for multiple comparisons, based on the GLMM results from Table S10.*

|  | Contrast | emmeans | SE | p-value |
| --- | --- | --- | --- | --- |
| Without each other | Pos - Ng | 0.321; 0.317 | 0.020 | 0.701 |
|  | Pos – Nt | 0.321; 0.324 | 0.020 | 0.839 |
|  | Ng– Nt | 0.317; 0.324 | 0.021 | 0.417 |
| With each other | Pos - Ng | 0.310; 0.324 | 0.019 | **0.032** |
|  | Pos – Nt | 0.310; 0.314 | 0.020 | 0.766 |
|  | Ng– Nt | 0.324; 0.314 | 0.022 | 0.157 |
| Negative | without each other – with each other | 0.317; 0.324 | 0.020 | 0.218 |
| Positive | without each other – with each other | 0.321; 0.310 | 0.022 | 0.053 |
| Neutral | without each other – with each other | 0.324; 0.314 | 0.022 | 0.074 |

*Table S12 Results from the GLMM model including valence, ROI and dyadic average scores (z-scores) as fixed and interacting factors, for the with each other condition. Results showed a significant effect of valence and of dyadic average scores.*

| Effect | *X*^2^ | Df | *p-value* |
| --- | --- | --- | --- |
| Valence | 1.808 | 2 | 0.405 |
| Dyadic average scores | 4.571 | 1 | **0.033** |
| ROI | 1.433 | 2 | 0.488 |
| Valence * Dyadic average scores | 0.539 | 2 | 0.764 |
| Valence * ROI | 2.815 | 4 | 0.589 |
| Dyadic average scores* ROI | 1.262 | 2 | 0.532 |
| Valence * Dyadic average scores * ROI | 7.923 | 4 | 0.094 |

*Table S13 Results from the GLMM model including valence, ROI and differences in subjective ratings (z-scores) as fixed and interacting factors, for the with each other condition. Results showed a significant effect of valence and a significant interaction between valence and differences in subjective ratings.*

| Effect | *X*^2^ | Df | *p-value* |
| --- | --- | --- | --- |
| Valence | 12.550 | 2 | **0.002** |
| Differences in subjective ratings | 1.454 | 1 | 0.228 |
| ROI | 1.419 | 2 | 0.492 |
| Valence * Differences in subjective ratings | 6.562 | 2 | **0.038** |
| Valence * ROI | 5.161 | 4 | 0.271 |
| Differences in subjective ratings* ROI | 2.275 | 2 | 0.321 |
| Valence * Differences in subjective ratings * ROI | 1.283 | 4 | 0.864 |

*Table S14 Summary of post hoc analyses concerning the correlation between IBS and differences in subjective ratings (z-scores), based on the GLMM from Table S13. A significant correlation was observed between IBS and differences in subjective ratings in the negative valence.*

| Valence | trend | SE | C.I. | p-value |
| --- | --- | --- | --- | --- |
| Positive | 0.011 | 0.015 | [-0.018 0.040] | 0.474 |
| Negative | -0.024 | 0.010 | [-0.043 -0.004] | **0.016** |
| Neutral | 0.024 | 0.022 | [-0.019 0.068] | 0.271 |

*Table S15 Summary of the post hoc contrasts analyses between the correlations of the interaction between IBS and differences in subjective ratings (z-scores), based on the GLMM from Table S13. No significant contrasts were observed.*

| Contrast | Estimate | SE | C.I. | p-value |
| --- | --- | --- | --- | --- |
| Positive-Negative | 0.035 | 0.018 | [-0.009 0.078] | 0.082 |
| Positive-Neutral | -0.014 | 0.027 | [-0.079 0.051] | 0.612 |
| Negative-Neutral | -0.048 | 0.024 | [-0.106 0.010] | 0.082 |

*Table S16 Results from the GLMM model including valence, ROI and dyadic average scores (z-scores) as fixed and interacting factors, for the without each other condition. Results showed no significant effects.*

| Effect | *X*^2^ | Df | *p-value* |
| --- | --- | --- | --- |
| Valence | 0.379 | 2 | 0.827 |
| Dyadic average scores | 0.009 | 1 | 0.923 |
| ROI | 3.377 | 2 | 0.185 |
| Valence * Dyadic average scores | 0.861 | 2 | 0.650 |
| Valence * ROI | 2.359 | 4 | 0.670 |
| Dyadic average scores* ROI | 2.040 | 2 | 0.360 |
| Valence * Dyadic average scores * ROI | 8.871 | 4 | 0.064 |

*Table S17 Results from the GLMM model including valence, ROI and differences in subjective ratings (z-scores) as fixed and interacting factors, for the without each other condition.*

| Effect | *X*^2^ | Df | *p-value* |
| --- | --- | --- | --- |
| Valence | 0.123 | 2 | 0.940 |
| Differences in subjective ratings | 3.254 | 1 | 0.071 |
| ROI | 3.399 | 2 | 0.183 |
| Valence * Differences in subjective ratings | 1.560 | 2 | 0.459 |
| Valence * ROI | 4.241 | 4 | 0.374 |
| Differences in subjective ratings* ROI | 0.495 | 2 | 0.781 |
| Valence * Differences in subjective ratings * ROI | 3.431 | 4 | 0.488 |

*Table S18 Results from the GLMM analyses with the mothers’ scores from IRI subscales – PD, PT and EC. The model revealed a significant effect of valence and of the interaction between valence and IRI – PD.*

| Factor | *X^2^* | Df | *p-value* |
| --- | --- | --- | --- |
| Valence | 10.713 | 2 | **0.005** |
| ROI | 1.393 | 2 | 0.498 |
| IRI - PD | 0.657 | 1 | 0.418 |
| IRI – PT | 0.007 | 1 | 0.936 |
| IRI – EC | 0.133 | 1 | 0.715 |
| Valence * ROI | 5.226 | 4 | 0.265 |
| Valence * IRI - PD | 6.150 | 2 | **0.046** |
| ROI * IRI – PD | 0.797 | 2 | 0.671 |
| Valence * IRI - PT | 1.374 | 2 | 0.503 |
| ROI * IRI – PT | 4.193 | 2 | 0.123 |
| Valence * IRI – EC | 0.355 | 2 | 0.837 |
| ROI * IRI – EC | 2.223 | 2 | 0.329 |
| Valence * ROI * IRI - PD | 3.330 | 4 | 0.504 |
| Valence * ROI * IRI – PT | 3.978 | 4 | 0.409 |
| Valence * ROI * IRI - EC | 1.953 | 4 | 0.744 |

*Table S19 Summary of post hoc analyses concerning the correlation between IBS and IRI - PD (z-scores). A significant correlation was observed between IBS and IRI – PD in the positive valence.*

| Valence | trend | SE | C.I. | p-value |
| --- | --- | --- | --- | --- |
| Positive | -0.020 | 0.009 | [-0.038 -0.002] | **0.032** |
| Negative | 0.017 | 0.013 | [-0.008 0.041] | 0.188 |
| Neutral | 0.002 | 0.015 | [-0.027 0.031] | 0.910 |

*Table S20 Summary of the contrasts between the correlations – between IBS and differences in subjective ratings (z-scores), based on the results presented in Table S17. A significant contrast between positive and negative valences was found for the correlation of IBS and IRI – PD.*

| Contrast | Estimate | SE | C.I. | p-value |
| --- | --- | --- | --- | --- |
| Positive-Negative | -0.036 | 0.015 | [-0.072 -0.001] | **0.049** |
| Positive-Neutral | -0.021 | 0.017 | [-0.062 0.019] | 0.313 |
| Negative-Neutral | 0.015 | 0.019 | [-0.031 0.061] | 0.434 |

*Table S21 Results from the GLMM model with the SSQ subscales – SBS and SHS. The model revealed a significant effect of valence and of the interaction between ROI and SBS.*

| Factor | *X^2^* | Df | *p-value* |
| --- | --- | --- | --- |
| Valence | 9.095 | 2 | **0.011** |
| SSQ – SBS | 0.041 | 1 | 0.839 |
| SSQ – SHS | 0.321 | 1 | 0.571 |
| ROI | 0.742 | 2 | 0.690 |
| Valence * ROI | 4.392 | 4 | 0.355 |
| SSQ - SBS * ROI | 6.416 | 2 | **0.040** |
| SSQ - SHS * ROI | 0.307 | 2 | 0.858 |
| Valence * SSQ - SBS | 2.109 | 2 | 0.348 |
| Valence * SSQ – SHS | 0.417 | 2 | 0.812 |
| Valence * ROI * SSQ - SBS | 3.579 | 4 | 0.466 |
| Valence * ROI * SSQ - SHS | 2.198 | 4 | 0.699 |

*Table S22 Summary of post hoc analyses concerning the correlation between IBS and SSQ - SBS (z-scores), based on the results from Table S20. A significant correlation was observed between IBS and SSQ - SBS in the frontopolar region.*

| ROI | trend | SE | C.I. | p-value |
| --- | --- | --- | --- | --- |
| Frontopolar | -0.029 | 0.013 | [-0.054 -0.003] | **0.026** |
| dlPFC | -0.001 | 0.013 | [-0.026 0.025] | 0.966 |
| TPJ | 0.013 | 0.014 | [-0.015 0.040] | 0.374 |

*Table S23 Summary of the contrasts between the correlations of the interaction between IBS and differences in subjective ratings (z-scores), based on the results from Table S22. A significant contrast between the frontopolar and TPJ regions was found for the correlation of IBS and SSQ - SBS.*

| Contrast | Estimate | SE | C.I. | p-value |
| --- | --- | --- | --- | --- |
| Frontopolar – dlPFC | -0.028 | 0.016 | [-0.066 0.010] | 0.118 |
| Frontopolar – TPJ | -0.041 | 0.017 | [-0.082 -0.001] | **0.043** |
| dlPFC - TPJ | -0.013 | 0.017 | [-0.054 0.28] | 0.440 |

*Table S24 Results from the GLMM model including only the right-handed dyads, with valence, social condition and ROI as fixed and interacting factors. Results showed a significant effect of social condition and a significant interaction between valence and social condition.*

| Effect | *X*^2^ | Df | *p-value* |
| --- | --- | --- | --- |
| Social Condition | 1.821 | 1 | 0.177 |
| Valence | 3.539 | 2 | 0.170 |
| ROI | 2.785 | 2 | 0.248 |
| Social Condition * Valence | 8.845 | 2 | **0.012** |
| Social Condition * ROI | 3.452 | 2 | 0.178 |
| Valence * ROI | 1.252 | 4 | 0.869 |
| Social Condition * Valence * ROI | 8.099 | 4 | 0.088 |

*Table S25 Post hoc results for the interaction between valence and social condition across all ROIs in the right-handed population, based on the GLMM results from Table S23. P values are FDR corrected. emmeans: 1st value corresponds to the first input in the contrast column.*

|  | Contrast | *emmeans* | SE | p-value |
| --- | --- | --- | --- | --- |
| Without each other | Pos - Ng | 0.322;0.320 | 0.014 | 0.973 |
|  | Pos – Nt | 0.322;0.322 | 0.015 | 0.997 |
|  | Ng– Nt | 0.320;0.322 | 0.016 | 0.973 |
| With each other | Pos - Ng | 0.313;0.322 | 0.013 | 0.047 |
|  | Pos – Nt | 0.313;0.320 | 0.015 | 0.403 |
|  | Ng– Nt | 0.322;0.320 | 0.016 | 0.973 |
| Negative | without each other – with each other | 0.320; 0.322 | 0.015 | 0.973 |
| Positive | without each other – with each other | 0.322; 0.313 | 0.016 | 0.047 |
| Neutral | without each other – with each other | 0.322; 0.320 | 0.016 | 0.973 |

Table S26 Spearman correlations between the absolute values of the differences between mother and child negative valence ratings and mother-child averaged arousal in the negative valence, mothers’ arousal in the negative valence, and children’s arousal during the negative valence for both social conditions. No correlation reached significance (p>0.10).

|  | Without each other | | | With each other | | |
| --- | --- | --- | --- | --- | --- | --- |
|  | Average mother-child arousal | Children’s arousal | Mothers’ arousal | Average mother-child arousal | Children’s arousal | Mothers’ arousal |
| Differences in mother-child ratings for the negative valence | 0.295 | 0.283 | 0.266 | -0.041 | -0.185 | -0.208 |

**Supplementary Figures**
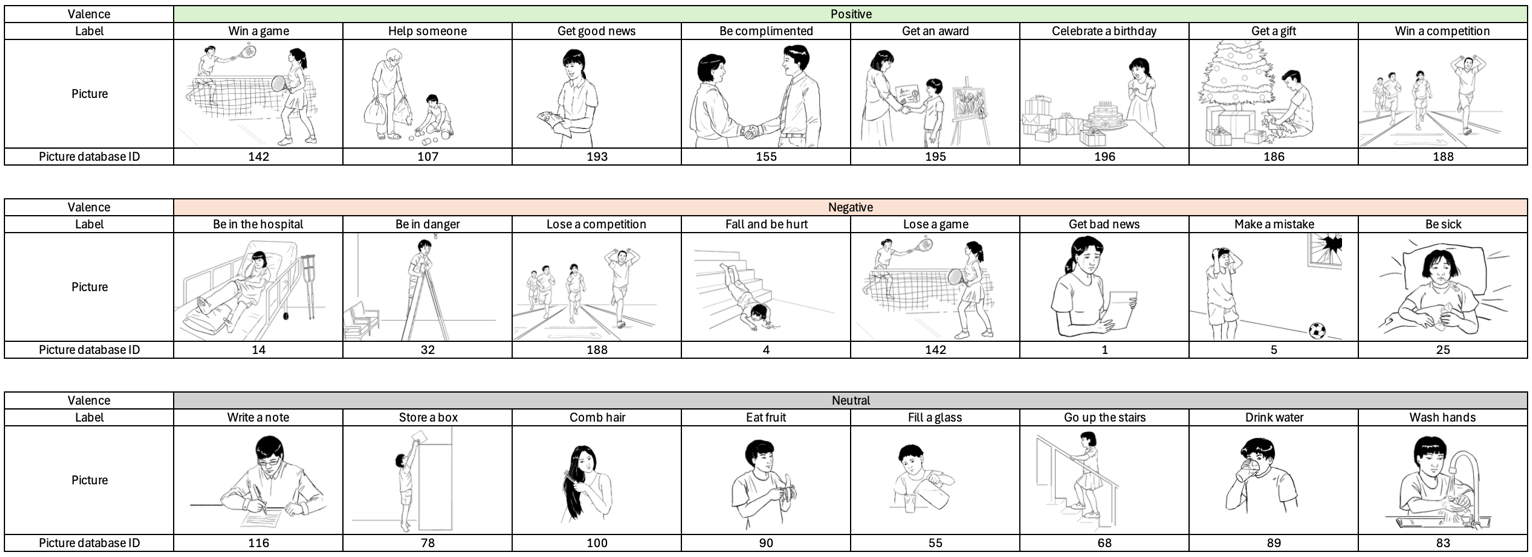


*Figure S1 Stimuli used for the emotional imagery task. There were eight different stimuli per valence, each one composed of a picture from the PiSCES database^7^ and a short label. The corresponding database ID can be found under each picture.*


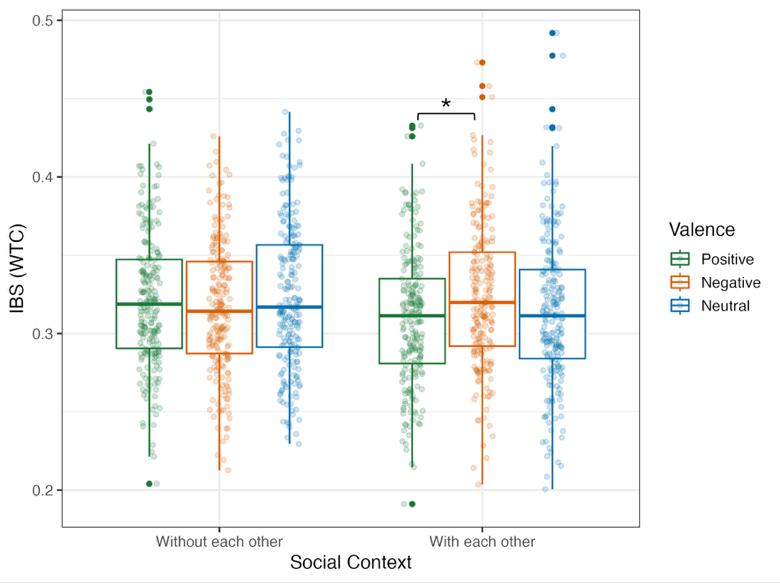


*Figure S2 IBS variation between valence and social condition in the right dlPFC. * p<0.05*


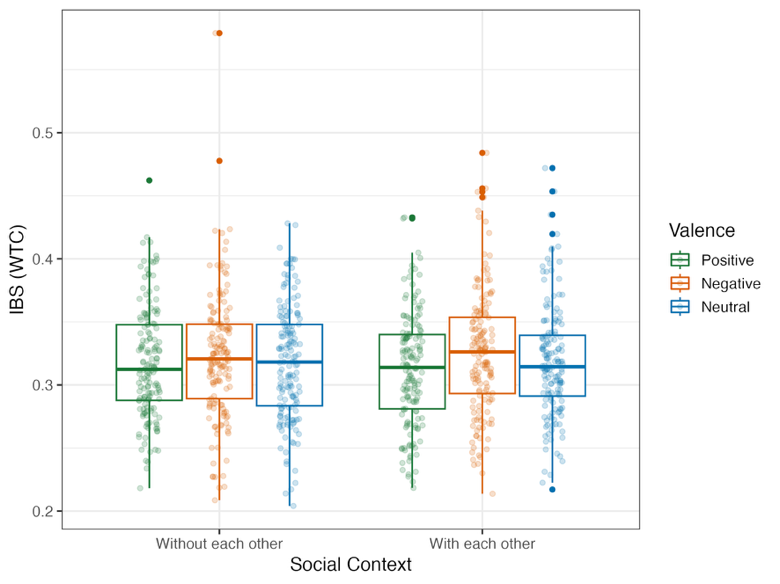


*Figure S3 IBS variation between valence and social condition in the right frontopolar region. No significant effects of valence, social condition or the interaction between the two.*

*
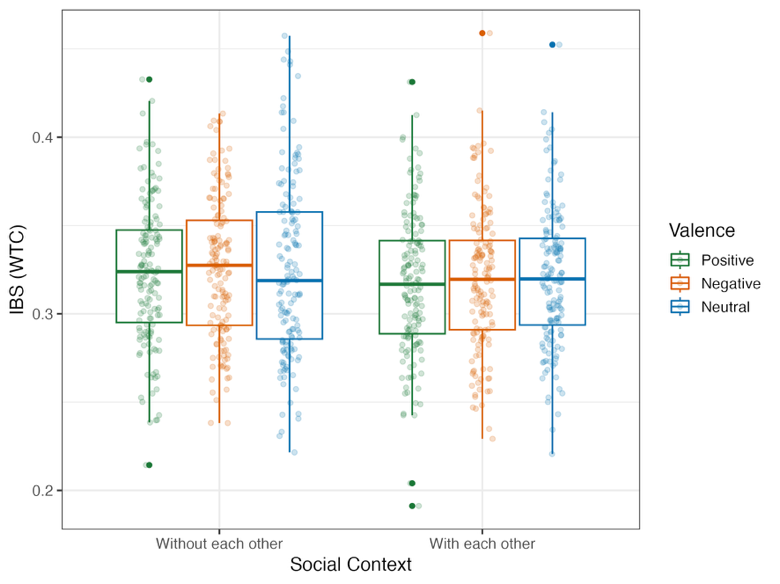
*

*Figure S4 IBS variation between valence and social condition in the right TPJ. No significant effects of valence, social condition or the interaction between the two.*

**References**

1. Feldman, R. The adaptive human parental brain: implications for children’s social development. *Trends Neurosci.* **38**, 387–399 (2015).

2. Lindquist, K. A., Wager, T. D., Kober, H., Bliss-Moreau, E. & Barrett, L. F. The brain basis of emotion: A meta-analytic review. *Behav. Brain Sci.* **35**, 121–143 (2012).

3. Long, M., Verbeke, W., Ein-Dor, T. & Vrtička, P. A functional neuro-anatomical model of human attachment (NAMA): Insights from first- and second-person social neuroscience. *Cortex* **126**, 281–321 (2020).

4. Nguyen, T. *et al.* The effects of interaction quality on neural synchrony during mother-child problem solving. *Cortex* **124**, 235–249 (2020).

5. Wang, S., Lu, J., Yu, M., Wang, X. & Shangguan, C. “I’m listening, did it make any difference to your negative emotions?” Evidence from hyperscanning. *Neurosci. Lett.* **788**, (2022).

6. Lian, T., Jiao, Z., Juan, S. & Zhang, P. Interpersonal brain synchronization in social pain contexts: an fNIRS-based exploration of empathy. *Soc. Cogn. Affect. Neurosci.* **20**, nsaf003 (2025).

7. Teh, E. J., Yap, M. J. & Liow, S. J. R. PiSCES: Pictures with social context and emotional scenes with norms for emotional valence, intensity, and social engagement. *Behav Res* **50**, 1793–1805 (2018).
